# The Oxidative Balance Orchestrates the Main Keystones of the Functional Activity of Cardiomyocytes

**DOI:** 10.1155/2022/7714542

**Published:** 2022-01-10

**Authors:** Michele Bevere, Caterina Morabito, Maria A. Mariggiò, Simone Guarnieri

**Affiliations:** ^1^Department of Neuroscience, Imaging and Clinical Sciences, University “G. d'Annunzio” of Chieti-Pescara, Chieti 66100, Italy; ^2^Center for Advanced Studies and Technology (CAST), University “G. d'Annunzio” of Chieti-Pescara, Chieti 66100, Italy

## Abstract

This review is aimed at providing an overview of the key hallmarks of cardiomyocytes in physiological and pathological conditions. The main feature of cardiac tissue is the force generation through contraction. This process requires a conspicuous energy demand and therefore an active metabolism. The cardiac tissue is rich of mitochondria, the powerhouses in cells. These organelles, producing ATP, are also the main sources of ROS whose altered handling can cause their accumulation and therefore triggers detrimental effects on mitochondria themselves and other cell components thus leading to apoptosis and cardiac diseases. This review highlights the metabolic aspects of cardiomyocytes and wanders through the main systems of these cells: (a) the unique structural organization (such as different protein complexes represented by contractile, regulatory, and structural proteins); (b) the homeostasis of intracellular Ca^2+^ that represents a crucial ion for cardiac functions and E-C coupling; and (c) the balance of Zn^2+^, an ion with a crucial impact on the cardiovascular system. Although each system seems to be independent and finely controlled, the contractile proteins, intracellular Ca^2+^ homeostasis, and intracellular Zn^2+^ signals are strongly linked to each other by the intracellular ROS management in a fascinating way to form a “functional tetrad” which ensures the proper functioning of the myocardium. Nevertheless, if ROS balance is not properly handled, one or more of these components could be altered resulting in deleterious effects leading to an unbalance of this “tetrad” and promoting cardiovascular diseases. In conclusion, this “functional tetrad” is proposed as a complex network that communicates continuously in the cardiomyocytes and can drive the switch from physiological to pathological conditions in the heart.

## 1. Introducing the Pillars of the Functional Activity in Cardiomyocytes

The heart, the first organ to be formed in the developing fetus and essential for life, is organized in a complex 3-dimensional tissue that is comprised of mechanically and electrically connected cardiomyocytes intimately coupled to capillary endothelial cells, fibroblasts, vascular smooth muscle cells, and macrophages [1].

The cardiomyocytes have an irregular structure and are smaller than skeletal muscle fibers with a diameter of about 10-15 *μ*m and a length within 50 *μ*m, and they usually have one nucleus. They are connected through their extremities by means of intercalated discs, specialized structures, in which cardiomyocytes' cellular membranes are highly jagged [2]. In intercalated discs, gap junctions and desmosomes play a key role. Gap junctions allow the electrical transmission of the action potential generated by cardiac pacemakers to the whole myocardium, while the desmosomes provide the mechanical link to ensure that the generated strength is propagated among cells and tissue matrix. Through this system, under physiological conditions, the frequency of heart beating is determined by the pacemaker cells of the sinoatrial node that give rise to spontaneous action potentials that spread through the conduction fibers of atria and ventricles triggering the contraction [3–6].

The peculiar feature of cardiomyocytes is the presence of organized clusters of myofibrils that contain myofilaments. The myofibrils have distinct and repeated microanatomical regions, the sarcomeres, which represent the basic contractile unit of the myocyte. The contraction process is triggered by an initial calcium ion (Ca^2+^) influx due to the opening of calcium channels located on the plasmatic membrane and activated by the electric signals. Thus, the interaction of the myofilament proteins, myosin and actin supported by other sarcomeric proteins, would not be possible without the crucial role of Ca^2+^ in the excitation-contraction (E-C) coupling mechanism [7, 8].

The activity of the sarcomeres is possible thanks to biochemical energy supplied and ensured by mitochondria. About of 1/3 of cardiomyocytes' volume is occupied by mitochondria; this explains why the heart is able to take up a major rate of oxygen from the blood than other tissues. In cardiomyocytes, the functional activity is characterized by a rhythmic contraction ensured by an interplay among myofilaments, intracellular Ca^2+^ variations (modulated by channels and pumps), and mitochondrial activity.

Other regulatory actors are emerging, such as the intracellular zinc ion concentration ([Zn^2+^]_*i*_). This aspect came out when the association between zinc deficiency/increase and the development of cardiovascular diseases was evident [9–14]. Indeed, intracellular Zn^2+^ plays a critical role in the modulation of the redox signaling pathway, thus affecting cardiomyocyte functions [10, 14]. This last aspect is important in the high-rate metabolic activity of cardiomyocytes, in which mitochondria are the main physiological producers of reactive oxygen species (ROS) that represent another intracellular signal. A failure in the mitochondrial function can lead to an uncontrolled increase in the levels of ROS, impairing the contractile function and damaging the cardiomyocytes [15].

In this scenario, sarcomeric proteins, intracellular Ca^2+^, intracellular Zn^2+^, and the metabolic machinery represent the main pillars of the functional activity in cardiomyocytes.

In particular, the intracellular redox balance, by regulating the functional status of proteins, acts as a common theme between the proteins of the contraction systems, of the metabolic activity, and of the Ca^2+^ and Zn^2+^ homeostasis, thus representing the cornerstone of cardiomyocyte function.

The aim of this review is to analyze the main components of the functional activity of cardiomyocytes and to outline their roles in a holistic scenario to underline the importance of the interplay and balance among contractile proteins, intracellular Ca^2+^ homeostasis, intracellular ROS management, and [Zn^2+^]_*i*_ movements in physiological and pathological conditions.

## 2. ROS Management: Eustress or Distress?

### 2.1. ROS and Their Sources

ROS are a group of different molecules with at least one oxygen atom and one or more unpaired electrons. They are divided into radical or nonradical species. The radical species contain one unpaired reactive electron in the outer orbit, while the nonradical species have two electrons.

Examples of radical species include superoxide anion (O_2_^•-^) and hydroxyl, peroxyl, and alkoxyl radicals, while those of nonradicals are hydrogen peroxide (H_2_O_2_), organic hydroperoxides, singlet molecular oxygen, hypochlorous acid, and hypobromous acid [16].

O_2_^•-^ has a key role in ROS signaling because it usually represents the first step in cascade reactions that produce other ROS [17]. O_2_^•-^ is generated by catalytic or noncatalytic reactions by electron transfer to molecular oxygen.

O_2_^•-^ has short life, because it is quickly converted in other ROS such as H_2_O_2_, for spontaneous dismutation or by superoxide dismutase (SOD) activity. When O_2_^•-^ combines with nitric oxide (NO), peroxynitrite forms [18].

H_2_O_2_ is a weak oxidant with a relatively long half-life, and it can act as a substrate to form extremely reactive species, such as hydroxyl radicals in the presence of endogenous iron by means of the Fenton reaction [19]. Hydroxyl radicals are also synthetized from electron exchange between O_2_^•−^ and H_2_O_2_ via the Haber-Weiss reaction [18].

In the heart, ROS modulate multiple physiological processes when present at low concentration, while an excessive ROS production can damage cellular components such as proteins, lipids, and nucleic acids determining oxidative stress linked to different pathological cardiovascular conditions [20].

**Figure 1:**
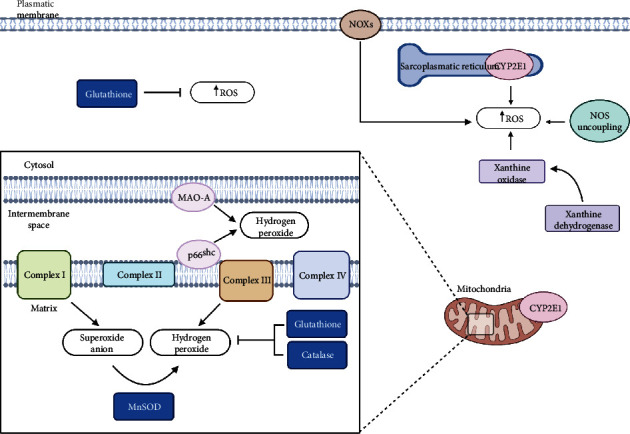
Intracellular sources of ROS and antioxidants.

The predominant sources of ROS in the heart cells are mitochondria, xanthine oxidoreductase (XOR), cytochrome P450 (CYP), NADPH oxidase (NOX), and nitric oxide synthases (NOS) [21] (Figure 1).

Mitochondria are the main cell supplier of ATP during the aerobic phase required for ordinary cell function and viability. The role of mitochondria is particularly relevant in tissue with high energy demands, as in the myocardium. Thus, about 30% of the heart's volume is occupied by mitochondria [22]. In adult cardiomyocytes, mitochondria are localized in three subcellular districts: the interfibrillar mitochondria, arranged in rows alongside the myofibrils; the subsarcolemmal mitochondria, placed in clusters just under the sarcolemma; and the perinuclear mitochondria, located nearby the nuclei [23].

Mitochondria are considered both the major producers of O_2_^•-^, generated during oxidative phosphorylation at the level of complexes I and III of the respiratory chain, and the main target of ROS-induced signaling. In mitochondria, the excessive ROS production can induce damage on lipids, proteins, and mitochondria DNA, with loss of mitochondrial membrane potential and with mitochondrial dysfunction resulting in cell death mechanisms [24].

A decrease in the rate of mitochondrial phosphorylation increases the electron leakage from the mitochondrial electron transport chain with the consequent production of O_2_^•-^, that is dismutated by mitochondrial SOD2 to H_2_O_2_.

H_2_O_2_ is also generated in mitochondria by redox activity of the growth factor adapter protein p66^shc^. This protein normally resides in the cytosol, but after exposition to UV radiation or treatment with oxidants, it is phosphorylated at the Ser36 level, translocates into the mitochondrial intermembrane space, oxidizes cytochrome c, and induces the production of H_2_O_2_, modulating the mitochondrial metabolism and the cellular response to oxidative stress. Surprisingly, the deletion of this protein leads to 30% prolongation of life span and increased resistance to ROS. Therefore, the pharmacology manipulation of p66^shc^ may be beneficial to fight chronic disease characterized by high ROS production, like cardiovascular diseases [25–28].

ROS can be generated also in the outer mitochondrial membrane by monoamine oxidase (MAO). MAO is a flavoenzyme that regulates the levels of catecholamine and serotonin through the oxidative deamination reaction in the heart. During this catabolic process, H_2_O_2_ and the corresponding aldehydes, as by-products, are formed. MAO has two isoforms: A and B, both expressed in the heart. It was shown that MAO expression and its ability to generate ROS increase with age and its role can be relevant in age-associated chronic disease, including hypertension, pressure overload, heart failure, and diabetes. The MAO-A overactivity elicits mitochondrial damage and myocardial degeneration in rodent models of pressure overload and diabetes, which can be effectively prevented by using MAO-inhibiting drugs [29].

MAO and p66^shc^ are considered key molecules of two independent pathways to produce H_2_O_2_, but they could be interconnected. Thus, p66^shc^, once phosphorylated, comes in the mitochondria, and it may induce the release of catecholamine by endogenous stores catabolized subsequently by MAO [30]. These pathways lead to increasing the ROS levels that can reduce the antioxidant defenses; this situation can trigger the development of heart failure [30]

The H_2_O_2_ detoxification in mitochondria occurs mainly through the glutathione (GSH) redox system, including the glutathione peroxidases that convert H_2_O_2_ to H_2_O and GSH reductases that restore intracellular GSH by reducing glutathione disulfide (GSSG), in addition to the presence of peroxiredoxins reducing equivalents of NADPH [31].

Peroxiredoxin 3 (Prdx3), that is the mitochondrial isoform, contributes to the elimination of almost 90% of mitochondrial H_2_O_2_ to maintain mitochondrial homeostasis [32]. Prdx3 has protective roles for the heart. Arimura et al. reported that the overexpression of Prdx3 in the mice protects the heart against left ventricular remodeling and failure after myocardial infarction [33]. The redox state of Prdx3 significantly changes during ischemia-reperfusion in the heart, and the reduction of the mitochondrial ROS by Prdx3 activity maintains cardiac function [34].

In mitochondria, H_2_O_2_ may be further reduced in H_2_O and molecular oxygen by catalase [32, 35]. Several studies demonstrated that catalase contributes to H_2_O_2_ detoxification in cardiac mitochondria, particularly when mitochondria are compromised [36]. In addition ion, in transgenic mice overexpressing catalase in the mitochondria, maximal lifespan was increased by 20%, and aging-associated cardiac pathology was significantly attenuated [37].

Besides these mitochondrial antioxidant defenses that ensure H_2_O_2_ elimination, aquaporins (AQPs) have been shown to modulate mitochondrial ROS generation. In particular AQP3, 5, 8, 9, and 11 are able to facilitate transmembrane diffusion of H_2_O_2_, and for this reason, these aquaporins are also defined “peroxiporins” [38, 39]. Therefore, in physiological conditions, H_2_O_2_ can move out of the mitochondria into the cytoplasm and function as a second messenger in signal transduction pathways [40].

Another source of ROS in the heart is represented by XOR, which catalyzes the oxidative hydroxylation of hypoxanthine to xanthine and xanthine to uric acid, the final two steps of purine metabolism in humans. These enzymes exist in two forms: xanthine dehydrogenase (XDH), which uses NAD^+^ as an electron acceptor, and xanthine oxidase (XO), which reacts with oxygen as an acceptor. Only XO produces ROS, catalyzing the oxidative hydroxylation of purine substrates with the formation of O_2_^•-^ and H_2_O_2_; under hypoxic and/or ischemic conditions, significantly more H_2_O_2_ is formed than O_2_^•-^ [41].

The basal expression of human XOR is low, but multiple factors can upregulate its transcription such as hormones, growth factors, inflammatory cytokines, and low oxygen tension [42]. Although there are discrepant reports in the literature, XO activity has also been demonstrated in the human heart [43].

Several studies demonstrated XOR upregulation in animal models of heart failure [44, 45] and in human dilated cardiomyopathy (DCM) [43, 46, 47]. XO inhibitors improve myocardial mechanical efficiency in both animals and humans with heart failure [48].

CYP450 enzymes belong to a family of heme proteins that catalyze the metabolism of a great number of endogenous and exogenous substrates. Cytochrome P450 2E1 (CYP2E1) is among the most active isozyme producing ROS and is in both endoplasmic reticulum and mitochondria. The expression levels of CYP2E1 increase significantly in human heart tissues under ischemic conditions and in animal models of DCM and hypertension. In these last models, CYP2E1 increase is often associated with the expression of several cellular markers of oxidative stress and apoptosis. The ablation of CYP2E1 in knockdown mice restores the level of these markers to a physiological range [49].

ROS can be generated at the plasma membrane level by NOX. These enzymes catalyze the production of O_2_^•-^, transferring an electron to oxygen from NADPH. O_2_^•-^ produced by NOXs rapidly dismutates to H_2_O_2_, and both ROS are released extracellularly and can reach the intracellular compartment by either simple diffusion, as in the case of H_2_O_2_, or via membrane-enclosed vesicles, as in the case of O_2_^•-^ [50].

NOX isoforms (NOX1-5 and Duox1/2) have a similar structure, containing at least six transmembrane domains and cytosolic flavin adenine dinucleotide (FAD) and NADPH-binding domains. NOX complexes are characterized by a distinguishing catalytic subunit and five variable regulatory phox subunits [51]. NOX2 and NOX4 are the main isoforms expressed in cardiac cells and are subjected to a distinct biochemical regulation. Activated NOX2 is predominantly expressed at the plasma membrane, whereas NOX4 is found in the cytosol although the precise location remains controversial. Numerous stimuli, including mechanical stretch, angiotensin II, endothelin-1, and tumour necrosis factor, alter the expression and activity of NOX proteins [46, 52, 53]. Experimental studies in animal models showed that NOX2 activation contributes to angiotensin II-induced cardiomyocyte hypertrophy, atrial fibrillation, and the development of interstitial fibrosis but may also positively modulate physiological excitation-contraction coupling. NOX2 contributes to myocyte death under stress situations and plays important roles in postmyocardial infarction remodeling, in part by modulating matrix metalloprotease activity. NOX4 is constitutively active at low level and induces protective effects in the heart under chronic stress, for example, by maintaining myocardial capillary density. However, high levels of NOX4 could have detrimental effects such as the development of cardiac hypertrophy and heart failure [54]. In contrast to NOX2, NOX4 has the ability to generate predominantly H_2_O_2_ instead of O_2_^•-^; this property may have important implications for interaction with NO signaling [55].

Therefore, there is significant evidence that NOXs contribute to the pathogenesis of hypertension. The NOX4 and NOX5 isoforms are considered the novel blood pressure-related genes, but there are necessary future studies to understand the mechanisms mediated by ROS levels and underlying hypertension [56].

Among ROS, also nitrogen reactive species (RNS) have to be considered. NO, generated by the corresponding NOS, is the representative molecule of RNS. The NOS has three isoforms (NOS1–3), and they catalyze the production of NO and citrulline using oxygen and L-arginine as substrates and tetrahydrobiopterin as a cofactor. Both NOS1 and NOS3 are constitutively expressed in the cardiovascular system. It is largely accepted that increased NO bioavailability can be considered a mean for cardioprotection against different cardiovascular diseases. However, NOS may be involved in the development of cardiovascular diseases, such as ischemia-reperfusion injury and cardiac hypertrophy. Thus, the tetrahydrobiopterin deficiency, due to its oxidation and/or reduced synthesis, can result in NOS uncoupling generating more ROS and less nitric oxide. This may lead to further oxidation of tetrahydrobiopterin, resulting in a positive feedback process that improves the oxidative damage with adverse consequences in the cardiovascular system [57].

### 2.2. Antioxidant Machinery

In physiological conditions, the cells activate their antioxidant defenses for neutralizing intracellular ROS.

In cardiomyocytes, the major antioxidant systems are SOD, catalase, glutathione peroxidase, and GSH.

SOD (cytosolic CuZn-SOD and mitochondrial Mn-SOD isoforms) initiates the detoxification of ROS by dismutation of O_2_^•-^ and converting it to H_2_O_2_. Both catalase and glutathione peroxidase further detoxify the H_2_O_2_ to H_2_O and oxygen. Glutathione peroxidase utilizes two GSH molecules as electron donors in the reduction of H_2_O_2_ to H_2_O, producing GSSG in the process. Once glutathione peroxidase oxidizes GSH to GSSG, GSH reductase can reduce GSSG back to GSH using NADPH, forming the GSH redox cycle [58].

The GSH is a peptide composed of glutamine, cysteine, and glycine, it is the most abundant thiol-containing peptide in eukaryotic cells, and it represents the most relevant endogenous antioxidant. The ratio of GSH/GSSG within cells is a measure of cellular oxidative stress where a decreased ratio is indicative of greater oxidative stress. In healthy cells, more than 90% of the total glutathione pool is the reduced form. The depletion of GSH rises the susceptibility to the rise of ROS and promotes the development of cardiovascular diseases [59].

Nonenzymatic antioxidant molecules taken from the diet are vitamin E, vitamin C, and vitamin K [60]. Polyphenolic compounds, including curcumin and resveratrol, activate Keap1/Nrf2/ARE (Kelch-like ECH-associated protein 1/NF-E2-related factor 2/antioxidant response element) pathways by enhancing the antioxidant defense [61]. Indeed, one of the antioxidant cellular responses is the activation of the complex formed by the Nrf2 and Keap1. In the cytosol, Nrf2 binds to its target Keap1 before the activation. Once the inducers (i.e., ROS) react with sulfhydryl groups of Keap1, Nrf2 dissociates and translocates into the nucleus, where it binds to and activates the ARE. It represents the promoter of transcriptional activation of antioxidant response element-containing genes, including glutathione, thioredoxin, and peroxiredoxin. In addition to this function, the Keap1/Nrf2/ARE pathway has been found to regulate genes involved in cell signaling, anabolic metabolism, autophagy, and organ development. Nrf2 and NF-*κ*B may fight in a coordinated fashion to maintain the redox balance. It was observed that the overexpression of Nrf2 limits the NF-*κ*B activity with anti-inflammatory effects, while the removal of Nrf2 induces an increased NF-*κ*B activity with proinflammation effects. Thus, Nrf2-knockout mice show high levels of inflammation and oxidative markers. In addition, diseases associated with inflammation (e.g., atherosclerosis, fibrosis, and lupus nephritis) are exacerbated when the Nrf2 pathway is inhibited [62, 63].

### 2.3. How ROS Modulate Cardiomyocyte Functional Behaviour

In healthy conditions, a low amount of ROS is produced during cellular metabolism, as in aerobic respiration or inflammation processes. A quantitative analysis showed that about 10-20% thiols of about 214,000 thiols in the cellular cysteine proteome are readily oxidized under aerobic conditions. These include enzymes, transporters, receptors, and transcription factor regulatory sites as well as allosteric and macromolecular interaction sites [64]. Thus, the physiological ROS signaling plays several roles contributing to orchestrating various complex processes such as cellular differentiation and proliferation, angiogenesis, adaption to the environment, and immune defense [65–69]. This low and physiological production of ROS is known as “oxidative eustress” [70]. In contrast, an altered balance between ROS generation and the endogenous antioxidant defence mechanisms that cause cellular dysfunction, protein and lipid peroxidation, and DNA damage has been called “oxidative stress” [71]. There are considerable experimental findings that demonstrate the oxidative stress which can lead to several cardiovascular diseases [72]. However, it has been observed that moderate production of ROS in mitochondria improves the systemic defense and induces an adaptive response that promotes longevity and metabolic health; this concept takes the name of “mitohormesis” [73].

ROS production also affects the mitochondrial dynamics. The processes of mitochondrial fusion, fission, biogenesis, and mitophagy determine mitochondrial morphology and size [74]. In particular, under mild oxidative stress conditions, the removal of defective mitochondria by mitophagy (the process of mitochondrial autophagy) reduces ROS levels and enhances cell survival. On the other hand, high oxidative stress promotes extensive mitochondrial fission (fragmentation of mitochondria) that ultimately leads to elevated ROS levels, loss of mitochondrial integrity, and apoptotic cell death [75].

The rise of intracellular ROS is associated with an increase in beating cardiomyocytes within embryo bodies, while the scavenging of ROS negatively promotes the cardiogenesis [76].

It has been observed that NOX4, the main isoform expressed in undifferentiated and neonatal cardiomyocytes, produces ROS and promotes the cardiogenesis. Conversely, the reduction of NOX4 or ROS scavenging suppresses the differentiation. In particular, the ROS, generated by NOX4, lead to the phosphorylation of p38 mitogen-activated protein kinase (MAPK) and to the consequent nuclear translocation of the cardiac transcription factor, the myocyte enhancer factor 2C (MEF2C). MEF2C is necessary to induce the cardiac phenotype and myofibrillogenesis. Thus, in addition to Ca^2+^-Ca^2+^/calmodulin-dependent protein kinase II (CaMKII), ROS are involved in the cardiogenesis. Both pathways have MEF2C as a target, and its translocation requires both Ca^2+^-CaMKII and ROS [77].

Another kinase regulated by ROS is the phosphatidylinositol 3-kinase (PI3K). PI3K is a critical downstream effector of *β*_1_ integrin signaling, which is activated by mechanical strain-induced ROS production and mediates the translocation of *β*-catenin into the nucleus, leading to increased connexin 43 and Nkx 2.5 levels required for cardiomyocyte differentiation. Conversely, antioxidants reduce levels of connexin 43 and Nkx 2.5 [78].

The intracellular Ca^2+^ overload represents a pathological signal in all types of cells including cardiomyocytes. This signal, as ADP/ATP depletion, leads to increased permeability of the outer mitochondrial membrane via activation of BAX and BAK proteins and the formation of mitochondrial permeability transition pore [79, 80]. This pore allows the release of the content of the mitochondria including the proapoptotic proteins such as apoptotic protease activating factor 1 and cytochrome c. Cytochrome c triggers the subsequent activation of the caspases inducing apoptosis and cell death [81]. The physiological opening of the mitochondrial permeability transition pore has been suggested to be a mechanism allowing the release of Ca^2+^ from overloaded mitochondria. The prolonged opening of this pore results in the overstated production of intracellular ROS, called “ROS-induced ROS release,” causing damage to lipids, proteins, and DNA [82, 83].

Mitochondria are critical players in the signaling pathways of cellular death and life [84]. Thus, several stresses can have both adaptive and maladaptive effects. The former effects contribute to life and resilience, while the latter ones lead to diseases. When adaptive effects are gradually depleted and maladaptive effects became dominant, the body shifts from physiological to pathological conditions.

## 3. The Proteins for Muscle Contraction: From Physiological to Pathological Conditions

**Figure 2:**
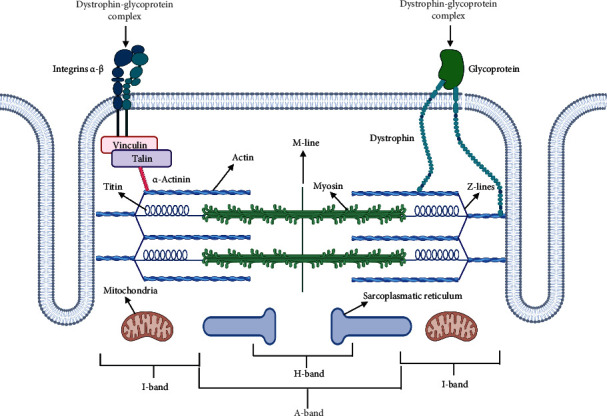
Structure of sarcomere with associated proteins.

In the striated muscles, the contractile unit is the sarcomere, and the correct assembly and regulation of its components are important in efficacious force development (Figure 2).

### 3.1. The Contractile Proteins

The main components of the sarcomeric structure are the thick and thin filaments mainly formed by myosin and actin, respectively.

The myosin filaments belong to a superfamily of myosins, a group of motor proteins formed of about 18 different classes and involved not only in muscle contraction but also in cell movements. The myosin present in cardiac muscle is known as myosin II. The myosin filaments are formed by two heavy chains (*α*- and *β*-MHCs) and two couples of light chains (MLCs) [85, 86]. The heavy chains of each molecule of myosin wrap on each other originating a double *α*-helical domain (tail), whereas on the N-terminal end, they form two globular regions (heads). The molecules of myosin are oriented in the opposite direction in the two halves of thick filaments with the tails toward the center region. Two light chains are associated with each globular head, for a total of 4 light chains [87].

The head of the myosin contains three different functional domains: the motor domain (sites involved in ATPase activity and for the binding to actin), the converter domain (which can be assimilated to a gearbox), and the lever arm. During the muscle contractile cycle, the motor domain binds to actin, products of hydrolysis (ADP and P_*i*_) are released, and the lever arm can rotate around the converter domain forming the actin-myosin cross-bridges [88]. Thirteen genes have been described for mammalian myosin heavy chain, including nine genes coding for sarcomeric myosin. These genes express different isoforms of the myosin heavy chain that characterize various muscle cells [89]. Different mutations, both in the heavy and in the light chain of myosin, are known to be responsible of pathological heart conditions underlying the importance of myosin in the cardiomyocyte functioning [90–92].

The thin filaments are composed mainly by globular monomers of G-actin that polymerizes to form filaments (F-actin). There are six genes in the human genome that codify different isoforms of actin that share more than 87% sequence identity: *α*-skeletal-, *α*-cardiac-, *α*-smooth-, *β*-cytoplasmic-, *γ*-smooth-, and *γ*-cytoplasmic-actin [93]. This protein is highly conserved and forms network with other cell structures (such as the plasma membrane) and is regulated by different actin-binding proteins. In muscle sarcomeres, actin is dynamically structured during assembly and even in mature myofibrils. Actin dynamics are regulated by enhancers (e.g., ADF/cofilin) and actin filament stabilizers (e.g., tropomyosin), and a malfunction of these regulators can be critical for assembly and maintenance of functional myofibrils [94].

There is evidence showing that knockout mice for *α*-cardiac-actin die either before or shortly after birth due to a loss of thin filaments in the sarcomere and consequently to cardiac failure [95]. As a primary component of the sarcomere, actin is necessary for proper sarcomere organization and function; indeed, several mutations in *α*-cardiac-actin result in different cardiac diseases, especially in cases of hypertrophic cardiomyopathy (HCM) and DCM [96–98].

In addition to the many genetic alterations, the proper functionality of the sarcomeric filaments can be affected also by posttranslational modifications, such as those due to ROS (or RNS). The effects of these reactive molecules can result in a direct chemical oxidation (or nitrosylation) of the contractile proteins, leading to changes in their structural conformation and functional activity. In particular, the cardiac myofilaments are affected by O_2_^•-^, mostly generated by the XOR system. These radicals are able to decrease the peak force expressed by the myofilaments, without altering their calcium sensitivity but compromising the cross-bridge kinetics and the ATPase activity [99]. In neonatal rat cardiomyocytes, the increase in ROS mediated by vasomotor peptides such as endothelin-1 [100] or angiotensin II [101] increased beta-myosin heavy chain gene expression inducing cardiomyocyte hypertrophy. Increased ROS levels act via the Ras/Raf/ERK pathway, and the administration of antioxidants restored these anomalies, underlying the important role of ROS in modulating the signal transduction involved in the cardiomyocyte hypertrophy. In addition, other proteins are sensible to ROS levels such as cardiac myosin-binding protein C (cMyBP-C), which is a thick filament assembly protein that interacts with both myosin and titin to regulate the cross-linkages of myosin in the A-band region. Indeed, the oxidation of cMyBP-C and cardiac troponin I cysteine residues was found to be associated with their hypophosphorylation. Accordingly, oxidized troponin I does not bind to troponin T, and the altered S-glutathionylation (an intracellular process directly regulated by the local redox status of the microenvironment) of cMyBP-C induces an impairment of cellular relaxation, with unaltered cellular Ca^2+^ dynamics. The reduction in cMyBP-C S-glutathionylation improves diastolic dysfunction, suggesting a strong link between S-glutathionylation and diastolic dysfunction [102].

Also, actin can be a target of the oxidants leading to contractile dysfunction and myocardial stunning. The S-glutathionylation of Cys-374 of actin is increased after postischemic reperfusion and causes decreased actin-tropomyosin interactions. In addition, glutathionylated actin showed structural alterations causing a decreased ability to polymerize compared to native actin. However, the administration of antioxidants restored physiological function of actin, supporting the idea that antioxidants may accelerate the recovery of contraction on postischemic reperfusion [103, 104].

### 3.2. The Regulatory and Structural Proteins

The efficacious interaction of the myofilaments is due to the presence of tropomyosin and troponin: the key regulators of cross-bridge cycling. The human tropomyosin family contains four genes (TPM1, TPM2, TPM3, and TPM4) that encode more than 40 alternatively spliced isoforms [105]. The *α*-tropomyosin is the main isoform expressed in cardiac and skeletal muscles. Indeed, the knockout of *α*-tropomyosin in mice leads to embryonic death [106, 107]. Tropomyosin molecules are folded end-to-end to form long strands along the actin filament following the long symmetry of the actin filament itself. One molecule of tropomyosin is combined with seven monomers of actin and binds to a complex of troponin [108].

The troponin complex is attached to the tropomyosin at regular intervals. It consists of three subunits: troponin-T (TnT), troponin-I (TnI), and troponin-C (TnC) [109]. TnC is the most studied isoform, and it contains Ca^2+^-binding EF hand motifs. The Ca^2+^ binding to the EF-motif leads to conformational changes allowing the N-terminal domain of TnC to interact with TnI [110]. TnI is the inhibitory subunit of the troponin complex, and it is able to block actomyosin ATPase activity in vitro [111]. The affinity of TnI to bind to actin depends on its conformational shift triggered by Ca^2+^ binding and consequently by Ca^2+^ concentration [112]. The third component of the troponin complex is TnT, composed of an IT-arm region, an inhibitory domain, a switch region, and a C-terminal mobile domain [113, 114]. The role of TnT is to anchor the troponin complex to tropomyosin [115]. The mechanism underlying the interaction between troponin and tropomyosin is correlated with Ca^2+^ levels. During diastole, when cytosolic Ca^2+^ level is low, the C-terminal domain of TnI strongly binds to actin, and tropomyosin blocks the actomyosin interaction (off-state). During systole, Ca^2+^ binds to the regulatory Ca^2+^-binding site of TnC, and this interacts with the C-terminal domain of TnI leading to its dissociation from actin (on-state); consequently, it induced a conformation change of the tropomyosin that shifts slightly in a position that discovers partially the myosin-binding site, increasing the probability of interaction between actin and myosin. Thus, this promotes the myosin-actin interaction and cross-bridge formation leading to the contraction [116–120].

To date, hundreds of mutations in all the members of the troponin family have been identified. In particular, the mutations that occur in genes coding for troponin and tropomyosin can lead to DCM, HCM, or restricted cardiomyopathy with different degrees of severity [121–125].

In an experimental model of coronary microembolization, a contractile impairment induced by both oxidation of tropomyosin and increase in TNF-*α* content has been observed. The oxidation of the cysteine-190 residue of cardiac tropomyosin modifies the protein interaction with TnT that plays a key role in the proper contractile function. On the other hand, TNF-*α* accumulation stimulates ROS production which, in turn, exacerbates both oxidative stress and inflammatory response. These effects were prevented by antioxidants [126].

Interestingly, tropomyosin appears more susceptible to oxidation than actin or desmin, although these proteins undergo oxidation in both in vitro and in vivo experimental models. Tropomyosin as well as actin has been shown to be a major target of oxidation through disulfide cross-bridge and S-nitrosylation formation; this results in contractile dysfunction that contributes to reducing left ventricular ejection fraction that limits cardiac output [103].

Among the proteins structuring the sarcomere, there is a group of numerous and various proteins with different functions such as connection, binding, and anchoring stabilization of myofilaments. In this context, two major representative proteins are described as follows: titin and nebulin.

Titin or connectin is a giant, stretchable protein that extends from the Z-line through the half I-band, over the thick filament, and ends at the M-line. It acts as a molecular spring, and it represents one of the main factors of the passive mechanical forces of the myofilaments. The force of titin arises from its extensible I-band region. Extension of the I-band segment of titin gives rise to part of the diastolic force of the cardiac muscle. In the I-band segment of cardiac titin, two main variants have been identified: N2BA and N2B. The ratio of N2BA and N2B isoform expression varies among and within species in different locations of the heart. The variation of this ratio may modify the level of passive tension for a given sarcomeric length. Cazorla and colleagues observed that the increased levels of N2BA lead to an increase in cell compliance. Conversely, the cardiomyocytes that express high levels of N2B are stiff. Thus, the diastolic properties of cardiomyocytes isolated from different species are not the same, but they vary due to the expression ratio of the titin isoforms [127]. Along the Z-line, A-band, and M-band, titin segments have primarily structural roles due to its binding to other main constituents of the sarcomere, including actin and *α*-actinin (Z-lines), myosin heavy chain protein and cMyBP-C (A-band), and myomesin (M-band) [128–130]. Titin provides binding sites for at least 30 other muscle proteins and is an important cue in cardiomyocytes [131].

A titin expression pattern that is shifted toward higher proportions of the N2BA isoform was found in end-stage human hearts, failing due to ischemia or DCM, differently from the human healthy hearts. The higher percentage of N2BA, as cited above, would lower sarcomeric stiffness, which could be a compensatory mechanism to contrast the condition of failing hearts [132, 133]. Truncating mutations in the gene that codifies titin have been identified in 20–25% of human patients with adult-onset DCM, which frequently has a genetic etiology [134]. DCM is not the only disease phenotype of titinopathy. Titin truncating variants are also found in women with peripartum cardiomyopathy, with prevalence like that observed in DCM [135]. Additionally, titin mutations have been reported in HCM [136], restrictive cardiomyopathy [137], and arrhythmogenic right ventricular cardiomyopathy [138].

Nebulin is part of a family of proteins highly conserved in vertebrates. It consists of module repeats that interact with troponin/tropomyosin complexes along actin thin filaments [139]. A single nebulin module interacts with one actin monomer, and each module repeat interacts with one thin filament regulatory complex (7 actin monomers : 1 tropomyosin :1 troponin complex) [140]. Nebulin is highly abundant in skeletal muscle, with very low amounts detected in the heart [141, 142]. If the role of nebulin is well known in skeletal muscle, where it acts as a “molecular ruler” modulating actin-myosin interaction and the length of thin filaments [143], little is known in cardiomyocytes. Cardiac-specific nebulin-knockout mice do not have shown an altered thin filament length indicating that nebulin has other distinct, yet undefined, roles in heart cells [144].

Titin is one of the major substrates for posttranslational modifications. Titin can be phosphorylated by a variety of kinases with different changes on passive mechanical properties of sarcomere. Indeed, phosphorylation of the N2B by cGMP-dependent protein kinase G (PKG) or cAMP-dependent protein kinase A (PKA) decreases titin stiffness, whereas phosphorylation of the PEVK-domain by protein kinase C (PKC) increases it [145]. In addition, both extracellular signal-regulated kinase 2 (ERK2) and CaMKII can phosphorylate different elements of titin resulting in a decrease in cardiomyocyte stiffness. In general, the beneficial effects of the phosphorylation of titin by several kinases (except for PKC) may include a reduction in myocardial diastolic stiffness and an improvement in ventricular filling [146].

Also, oxidative stress affects this protein by triggering disulfide bridge formation in the N2B thus decreasing titin extensibility and increasing titin-based passive tension, as observed in isolated human cardiomyofibrils. Consequently, titin stiffening could thus contribute to the alterations in myocardial mechanic alterations associated with oxidative stress, which often accompanies aging or heart failure. It has been shown that thioredoxin, which catalyzes disulfide bond formation and isomerization, reduces the stiffness of isolated human cardiomyofibrils [147]. Another direct oxidative stress-related effect on titin-based stiffness is the S-glutathionylation of cryptic cysteines in the Ig-domains of the elastic I-band region, leading to decreased refolding of the Ig-domains. This S-glutathionylation causes the titin spring to become longer, thereby contributing to a decrease in cardiomyocyte passive stiffness in a reversible way [148]. Overall, posttranslational modifications alter titin-based myocardial passive stiffness representing an attractive target for therapeutic treatment in common forms of heart failure, especially heart failure with preserved ejection fraction. However, the role that oxidative balance plays in dynamic stiffness regulation of titin in vivo should be further explored [149].

The sarcomere is not a separate unit inside the muscle fibers, but it is an integral part of the complex arrangement that works in concert with the extracellular matrix (ECM) and sarcolemma to perform a functional contraction and strength generation. The cardiac ECM is important for the structural integrity of the heart providing mechanical stiffness. Mainly, the cardiac ECM is composed of collagens, glycoproteins (e.g., fibronectins, elastin, and laminins), and proteoglycans [150].

The sarcomere is tethered to the sarcolemma by a cytoskeletal assembly named costamere. It is a macromolecular protein structure arranged in line with the Z disk, composed of the vinculin-talin-integrin system and the dystrophin-glycoprotein complex. The costamere links the cytoskeleton to the ECM, transmitting force from the sarcomere to the ECM (“inside-out”) and conversely from the ECM to the myocytes (“outside-in”). The costamere couples the force generated by the sarcomere with the sarcolemma, and it is considered a weak point of the cardiac muscle that, if compromised, can lead directly to the onset of important cardiomyopathies [151]. Thus, we can consider sarcomeres highly specialized forms of the actin cytoskeleton, the other proteins can be defined as a dynamic protein network of “sarcomeric cytoskeleton” [128].

Vinculin and talin are tethered to the costamere via their interaction with integrins and serve as adaptor proteins [152]. Integrins orchestrate multiple functions including adhesion, ECM organization, signaling, survival, and proliferation. In cardiomyocytes, integrins are mechanotransducers, translating mechanical to biochemical information [153].

Although integrins do not possess their own enzymatic activity, they are potent bidirectional signaling receptors, converting events outside the cell to intracellular signals and vice versa.

This means that when ECM ligands bind to the extracellular integrin domains, intracellular signaling occurs through a process commonly termed “outside-in” signaling. Ligand binding implies a wide range of intracellular signal proteins including integrin-linked kinase, vinculin, and talin. Experiments in vitro have shown that the binding of integrins to ECM resulted in integrin clustering, followed by focal adhesion protein complex formation, actin polymerization, and finally actin-myosin formation, thus providing rigidity to the cell and a mechanosensitive link between the extracellular and intracellular environments [154].

In contrast to these extracellular events, those happening within the cell can also include the direct or indirect binding of the integrin cytoplasmic domain, enabling integrin activation. This process is known as “inside-out” signaling because events inside the cell trigger integrins' conformational changes altering their ECM-binding characteristics [155].

The signals activated during cell attachment have been shown to be influenced by integrin-triggered production of ROS from several sources. ROS derived from mitochondria as well as from NOXs were reported to have important roles in integrin-mediated attachment, spreading, and the associated changes in the cytoskeleton [156–158].

Acting as second messengers, ROS produced by integrins upon ECM can regulate cytoskeleton dynamics through both direct and indirect patterns. ROS can directly activate Src tyrosine kinase in response to integrin receptor engagement or can indirectly act on cytoplasmic target proteins as low molecular weight phosphotyrosine phosphatase or cytoskeletal actin. The latter, undergoing S-glutathionylation (cysteine 374), leads to cytoskeleton organization and cell spreading. The impairment of this glutathionylation, due to GSH depletion, leads to a rounded shape and a ring-like actin cytoskeleton [159].

Dystrophin is the primary element of the dystrophin-glycoprotein complex that is the second major component of the costamere and connects the sarcolemma to the ECM [160].

Dystrophin is made up of two calponin homology domains. The NH_2_-terminal regions of dystrophin and utrophin bind to the cytoskeletal actin, acting as the intracellular anchor, whereas the COOH-terminal regions bind to a group of proteins (mainly laminin) anchored to the cell membrane. Thus, this scenario includes a new growing family of cell-anchoring molecules [161].

Many mutations of a gene encoding dystrophin promote the development of the Duchenne and Becker muscular dystrophies. The Duchenne muscular dystrophy has been extensively studied using, for example, the mdx mouse [162]. The cardiac phenotype of mdx mice replies to the human Duchenne muscular dystrophy one. This animal model shows a cardiac muscle deterioration that worsens with age leading to fibrosis, decreased cardiac efficiency, and dysfunction up to death [163].

It is well known that the hearts of mdx mice exhibit several cellular abnormalities including loss of sarcolemmal integrity, increased calcium influx, mitochondrial alterations, and an increase in ROS, which are reduced by the administration of antioxidants in vitro [164]. In this study, it has been observed that there is an increase in expression and activation of NOX in mdx mice that may induce high ROS levels. The cause of this increased activity of NOX may be likely both the increased of muscle stretch and of caveolin-3 (due to the absence of dystrophin) which localizes with NOX on membranes. Overall, the increased oxidative stress may account for the changes in Ca^2+^ handling, myofilament dysfunction, and inflammation as in DCM observed in Duchenne muscular dystrophy [165]. Other experimental evidence showed, in the heart of mdx mice, an increased susceptibility to opening of mitochondrial permeability transition pore, enhanced activation of cell death signaling, and mitochondrial oxidative stress, which contribute to cardiomyocyte damage. Interestingly, the inhibition of the mitochondrial permeability transition pore in the heart of mdx mice reduced cytosolic and mitochondrial Ca^2+^ alterations and mitochondrial damage ameliorating cardiopathy in older mdx mice [166].

In this regard, therefore, the “integrin signals” can be considered to be composed of separate sets of reactions triggered by different types of integrin stimulation. Mitochondrial and extracellular ROS have specific and distinct effects on integrin signals induced by cell attachment and mechanical stretching.

## 4. Intracellular Ca^2+^: The Driving Force of Cardiac Contraction

Ca^2+^ is a key element critical for many biological functions. Despite its importance in live organisms, Ca^2+^ is present at very low concentrations, in the order of 10^−3^M in extracellular fluids. Nevertheless, the concentrations of intracellular free Ca^2+^ in resting conditions are around 10^−7^M, four orders of magnitude lower than that present outside the cells. This difference provides the potential “engine” for the ready import of Ca^2+^ into cells, where it performs its function as a second messenger. Intracellular Ca^2+^ acts as a universal and versatile second messenger that regulates vital biological processes, including cell contraction, synaptic transmission, hormone secretion, cell growth, cell death, and even more [167]. A variety of extracellular factors promotes, via Ca^2+^ channels, the fluxes of Ca^2+^ either from outside across the cell membrane or from intracellular stores into the cytoplasm. The contraction process in skeletal and cardiac muscle cells is a consequence of changes in intracellular Ca^2+^ concentration as a response to electrical depolarization signals. For these reasons, intracellular Ca^2+^ represents the link between electrical signals and the mechanical response of contraction in cardiomyocytes able to push the blood. In cardiomyocytes, there are defined groups of “main players” involved in handling Ca^2+^ signals to allow the main two phases of the heart cycle: systole and diastole. They are voltage-operated Ca^2+^ channels (VOCCs), ryanodine receptors (RyRs), and calcium pumps/transporters [168].

Striated muscle has a peculiar type of membrane, the sarcolemma, containing the transverse tubules (T-tubules). T-tubules are invaginations of the sarcolemma and provide a major surface for hosting several ion channels, ion transporters, and pumps involved in Ca^2+^-handling. Thus, the main function of T-tubules is the regulation of E-C coupling. In cardiomyocytes, the L-type VOCCs (Ca_v_1.2, characterized by a long-lasting inactivation currents), localized in T-tubules, open after depolarization allowing the influx of Ca^2+^ ions letting their positioning in proximity to RyRs placed on the membrane of the terminal cisternae of the sarcoplasmic reticulum (SR). Consequently, Ca^2+^ ions trigger the opening of RyRs inducing the “Ca^2+^-induced Ca^2+^ release” (CICR) sequence and consequently triggering the contraction. In cardiac muscle cells, one T-tubule and one terminal cisterna form a dyad, while the Ca_v_1.2 channels and RyRs, strictly close, constitute the calcium-releasing units (CRUs). In these structures, the Ca^2+^ transients are initiated following the action potential, the depolarizing stimulus, that opens the VOCCs thus initiating the CICR [169, 170].

Therefore, VOCCs, RyRs, and pumps can be considered the central players controlling cardiac Ca^2+^ fluxes and their concentration inside the cells. In addition, there is a network of a great number of accessory proteins able to influence the regulation of cardiac Ca^2+^ signal. Therefore, subtle changes in the components of the regulatory machine can induce several and/or severe consequences on the function and phenotype of cardiomyocytes.

**Figure 3:**
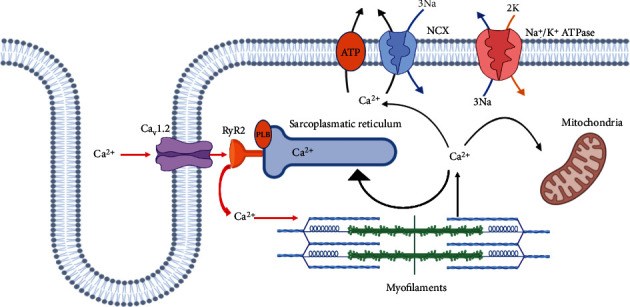
Input and output of Ca^2+^ signals (red and black arrows, respectively).

Thanks to experiments carried out by Ringer, more than 100 years ago, we know the important role of Ca^2+^ in E-C coupling [171]. The pacemaker cells undergo spontaneous depolarization and thereby generate a propagating action potential that involves each cardiomyocyte. During an action potential, the membranes of cardiomyocytes are depolarized, leading the opening of Ca_v_1.2 and consequently Ca^2+^ entry that is confined in the dyad space. This local Ca^2+^ rise is called “Ca^2+^ sparklet,” and it is not sufficient to promote the cardiac contraction but triggers the opening of the RyRs on SR and consequently the release of Ca^2+^ from SR, in the CICR process [172]. This final cytosolic Ca^2+^ increase, called sparks, is able to induce cardiac contraction. During one single action potential, thousands of Ca^2+^ sparks are activated simultaneously by charges of the Ca^2+^ sparklet [167, 173]. After RyR activation, Ca^2+^ spread outside to the dyadic space to trigger the contractile machinery, promoting cell shortening to provide the necessary force to pump blood. At the end of action potential, intracellular Ca^2+^ returns to resting levels, mainly corresponding to the diastolic phase, thanks to calcium pumps, in preparation to the following depolarization event (Figure 3).

In cardiomyocytes, the Ca_v_1.2 channels mediating the influx of Ca^2+^ in response to depolarization are composed of a pore-forming *α*_1_ subunit of ∼190–250 kDa and several auxiliary subunits including *α*_2*δ*_, *β*, and *γ*. The *α*_1_ subunits expose the binding sites for different regulators and molecules, whereas the other subunits contribute to trafficking, anchorage, and regulatory functions. The kinetic of these channels is characterized by different possible states of gating: mode 0 in which channels are not or rarely open; low mode 1, in which there is a low probability that channels will open, but just for a short period; and high mode 2 characterized by frequent long-lasting openings [174].

The inactivation of Ca_v_1.2 channels is mediated by both repolarization and Ca^2+^ itself. The intracellular concentration of Ca^2+^([Ca^2+^]_*i*_) rises can both amplify the Ca^2+^ influx, in an event called calcium-dependent facilitation, and block a further ionic rise by a Ca^2+^-dependent inactivation. The balance and timing between Ca^2+^-dependent facilitation and inactivation of Ca_v_1.2 play a key role in regulating the magnitude of Ca^2+^ influx [175].

These mechanisms involve Ca^2+^ binding to calmodulin (CaM) and the protein interaction with the IQ domain in the C-terminal tail of Ca_v_1.2 channels [176]. CaM is a small *α*-helical protein that binds to Ca^2+^ leading it toward the several targets. CaM is constituted by 148 amino acids composed of N- and C-terminal lobes, each of these contains two Ca^2+^-binding EF hands. So, one molecule of CaM can bind to four Ca^2+^ ions. These two lobes bind to Ca^2+^ in a different way: one with high affinity for Ca^2+^ in the C-terminal site and the other with low affinity in the N-terminal site. Therefore, CaM can detect both local and global Ca^2+^ levels with a highly efficient system thus regulating also Ca_v_1.2; this process is known as “the calmodulation” [177]. When Ca^2+^ flows in the cardiomyocytes via Ca_v_1.2 channels, Ca^2+^ binds to CaM inducing the conformational changes from Apo-CaM to Ca^2+^-CaM; in this state, this complex can interact with several intracellular targets modulating their activity [178, 179]. Ca_V_1.2 channels in the heart muscle are arranged in clusters at the cell membrane. When the calcium ions enter the cell, they bind to CaM that in turn binds to the IQ domain at the C-terminal of Ca_v_1.2 channels, inducing channels' interaction. The physical clustering of Ca_V_1.2 channels enables them to work in a cooperative way; this ensures the opening and closing states of Ca_v_1.2 channels in concert [180].

Another important player in the Ca^2+^ handling is RyR. There are three human isoforms of RyRs (1, 2, and 3) located on SR. RyR2 is the major isoform in the heart, and it has a delicate role in E-C coupling. RyR2 is a homotetrameric channel with a morphology resembling four-leaf clover or mushroom, formed by the transmembrane domains and cytoplasmic domains. The latter extremity protrudes in the narrow space of about 15 nanometers from Cav1.2 channels [181, 182]. Ca^2+^ binding to RyR2 causes its conformational changes with the twisting of the transmembrane regions and the consequent opening of the channel pore in a mechanism similar to that of a camera iris [183]. RyR2 channels are modulated directly and indirectly by the Cav1.2 channels and by various ions, small molecules, and proteins, e.g., Ca^2+^, calstabin2, CaM, CaMKII, calsequestrin, triadin, junctin, and PKA [181].

Even if RyR2 is the main component able to release calcium from SR, the regulatory proteins are fundamental for its functioning. If the opening of RyR2 promotes the calcium release making the systole possible, during the resting phase, the binding of calstabin2 (also known as FK506-binding protein (FKBP12.6)) to RyR2 helps to keep close the channel to limit the leak of SR-Ca^2+^ into the cytosol [184]. CaM also regulates RyR2 by a direct binding decreasing the probability of channel opening [185].

CaMKII is another regulatory protein that has four isoforms *α*, *β*, *γ*, and *δ*, each isoform being encoded by a separate gene. The main CaMKII isoform present in the heart is the *δ* one [186]. CaMKII*δ* is a dodecameric enzyme with a N-terminal catalytic domain, a C-terminal association domain, and a middle autoregulatory domain including also a Ca^2+^-CaM binding site [187]. The activation of CaMKII*δ* is modulated in a Ca^2+^ dose-dependent mechanism and also by Ca^2+^ spark frequency, amplitude, and duration [188]. When CaMKII*δ* is inactivated, the catalytic domain is sterically blocked by the regulatory domain (autoinhibitory state). When Ca^2+^-CaM binds to the CaMKII*δ* regulatory domain, it induces the enzyme conformational change that leads to subsequent autophosphorylation. This promotes the activity of CaMKII*δ* also after the dissociation from Ca^2+^-CaM [189].

Under physiological conditions, CaMKII orchestrates a response that is acutely adaptively dynamic and functional, while it is found dysregulated in structural heart disease [190].

Indeed, CaMKII is poised to respond to prolonged Ca^2+^ transients (as in Ca^2+^ remodeling) and rapid Ca^2+^ fluxes (as in tachycardia) due to its molecular structure and function [187]. On the other hand, CaMKII can also affect cellular Ca^2+^ homeostasis by regulating Ca_v_1.2 channels and SR-Ca^2+^ uptake and release. Thus, CaMKII has appropriate subcellular localization and is endowed with structural and functional characteristics both to contribute to intracellular Ca^2+^ homeostasis alterations and to respond to these changes by increasing its activity [191]. At increased heart rates, the increase in time-averaged [Ca^2+^]_*i*_ leads to the activation of CaMKII that allows the binding and the phosphorylation of two candidate sites on the Ca_v_1.2 channel: the pore-forming *α*1C subunit and the auxiliary *β*2 subunit, determining an increase in Ca^2+^-entry via Ca_v_1.2. Thus, CaMKII*δ* causes Ca_v_1.2 to enter a high activity gating mode (mode 2) [192]. In addition, CaMKII*δ* can phosphorylate and open RyR2 causing a rise of SR-Ca^2+^ release. This produces a higher Ca^2+^ transient that activates CaMKII*δ* itself, increasing resting SR-Ca^2+^ release or leak [193]. In brief, the phosphorylation of CaMKII*δ* determinates intracellular Ca^2+^ overload triggering a delayed afterdepolarization, and this mechanism develops premature ventricular contractions that can lead to the development of arrhythmias. In polymorphic ventricular arrhythmia, CaMKII expression is increased and CaMKII inhibitory agents reduced or prevented arrhythmias in mouse models [194].

A major part of Ca^2+^ in SR (50-90%) is bound to the low-affinity Ca^2+^ buffering protein calsequestrin. There are two isoforms of this protein: calsequestrin1, located in fast skeletal muscle, and calsequestrin2 in heart tissue. Calsequestrin2 plays a key role in CICR and E-C coupling. As a buffering protein, calsequestrin2 delivers Ca^2+^ nearer the luminal regulatory sites of RyR2, influencing consequently the process of Ca^2+^ release. In addition, calsequestrin2 modulates the RyR2 state reducing its opening probability by interacting with triadin and junctin, two other regulatory proteins [195].

Triadin is an integral membrane protein in junctional SR vesicles. The cardiac isoform is triadin-1 [196]. Two main functional roles have been described for triadin-1. This protein acts as a bridge anchoring calsequestrin2 near the junctional zone of SR. In this way, triadin-1 allows indirectly Ca^2+^ buffering by calsequestrin2 in SR [197]. The triadin-1 modulates Ca^2+^ channel activity by increasing the opening probability of RyR2 [198]. Indeed, experimental evidence revealed that the overexpression of triadin-1 enhances the activity of SR-Ca^2+^ release, with a consequent SR-Ca^2+^ leak and proarrhythmic Ca^2+^ spikes.

Junctin is a small protein that binds to calsequestrin2 in junctional SR and RyR2. Indeed, the triadin-1-junctin complex binds to luminal RyR2, increasing its opening probability. This agonist effect is blocked by calsequestrin2 that binds to these two proteins and reduces this activation. Calsequestrin2 acts as a luminal Ca^2+^ sensor that inhibits RyR2 at low luminal [Ca^2+^], whereas triadin-1 and junctin may be needed for a physical bridge between calsequestrin2 and RyR2. The triadin-1-junctin complex stabilizes RyR2 in an open state and, consequently, balances the inhibitory regulation mediated by calsequestrin2 [199].

PKA is a family of enzymes whose activity is dependent on cellular cyclic AMP (cAMP) levels. Protein-kinase A plays several functions in the cell, among which is the modulation of Ca_v_1.2 and RyR2. In response to stimuli that involve the activation of *β*-adrenergic receptors, the intracellular levels of cAMP increase. Consequently, protein-kinase A is activated and, in turn, it can phosphorylate both Ca_v_1.2 and RyR2, enhancing the activity of these channels. Protein-kinase A has a positive inotropic response on Ca_v_1.2 channels, increasing both their number and opening probability [200]. Indeed, protein-kinase A phosphorylates also RyR2 and, consequently, induces the separation of calstabin2, leading to an increase in opening probability of these channels [201].

Given the importance of these proteins and channels in controlling CICR and E-C coupling, it is not surprising that the acquired or genetic defects on these elements lead to cardiac pathological states. Considering the Ca^2+^ channels' roles in the contraction process, mutations of the gene that codifies Ca_v_1.2 channels are associated with a severe variant of long QT syndrome, the Timothy syndrome [202]. The disarray in the calstabin2-RyR2 interaction can cause arrhythmia in catecholaminergic polymorphic ventricular tachycardia [203], confirmed by results obtained in calstabin2-deficient mice [204]. In addition, the polymorphic catecholaminergic ventricular tachycardia is known to arise from mutations in RyR2 [205], calsequestrin2 [206], and CaM [207, 208]. All these mutations are now recognized to increase RyR2 activity, highlighting the important role of SR-Ca^2+^ release in cardiac rhythm.

Moreover, it was seen that the increased CaMKII activity in heart failure can contribute to reducing SR-Ca^2+^ content and systolic function and causes diastolic SR-Ca^2+^ leak and Ca^2+^ current changes that may be arrhythmogenic [209]. Thus, diastolic SR-Ca^2+^ release events, if sufficient in amplitude, can trigger spontaneous Ca^2+^ waves at the cellular level. Indeed, intracellular Ca^2+^ overload is associated with an increased propensity of spontaneous SR-Ca^2+^ release, which can lead to a delayed afterdepolarization because of the transient inward current carried by the Na^+^-Ca^2+^ exchanger (NCX, in the Ca^2+^ extrusion mode) [210]. In mouse models, arrhythmias were significantly suppressed by an inhibitory agent targeting endogenous CaMKII*δ* [211]. In addition, CaM and CaMKII*δ* can be involved in hypertrophic cardiomyopathy. Gruver and colleagues reported that targeted overexpression of CaM induces a proliferative effect and hypertrophic growth of cardiomyocytes in transgenic mice [212]. In addition, CaMKII*δ* has a central role in pathological hypertrophic pathways [213]. It has been reported that hypertrophic stimulation increases CaMKII*δ* expression in cardiomyocytes, leading to histone H3 phosphorylation and chromatin remodeling. This phosphorylation event allows the activation of prohypertrophic fetal cardiac genes [214]. It is important to mention that the overexpression of triadin-1 and junctin in animal models induced proarrhythmic Ca^2+^ transients and decreased levels of calsequestrin2 [198, 215].

Several Ca^2+^ handling proteins are regulated by ROS causing mostly alteration in Ca^2+^-homeostasis associated with direct modification of target proteins (i.e., ion channels and transporters) as well as activation of serine/threonine kinases. Voltage-dependent Ca^2+^ channels are redox sensitive, and the pore-forming unit (*α*_1C_ subunit) of Ca_v_1.2 contains 48 cysteines with approximately 38 cysteines in sites that are accessible to modification by glutathionylation. In this respect, the modifications induced through the variations of the redox state (increases or decreases) can affect activity, expression, and open-time probability of these channels, as well as trafficking [216, 217]. Application of H_2_O_2_ increased ionic currents in cells expressing human cardiac L-type *α*1 subunits in a voltage-dependent manner. Indeed, the exposure to H_2_O_2_ or GSSG in vitro results in the S-glutathionylation of Ca_v_1.2 through direct modification of thiol groups on the channel leading to an increase in its open probability and, consequently, in Ca^2+^ influx [218]. Extracellular oxidation of the L-type calcium channel leads to an increase in mitochondria-derived O_2_^•-^. This increase is reversible and is associated with increased intracellular Ca^2+^ and influx of Ca^2+^ into the mitochondria as a result of an increase in basal Ca^2+^ currents. The observed effect appears to be specific as other proteins involved in calcium handling are not affected (e.g., RyR) [219]. However, other authors demonstrated a reduction in L-type calcium currents when guinea pig ventricular myocytes were treated with oxidants [220]. These controversial results may be a consequence of free radical interaction on different components of the machinery for the management of Ca^2+^.

Also, CaMKII, PKC, and PKA can activate Ca_v_1.2 in a ROS-dependent mechanism [174]. The exposure to H_2_O_2_ induced an increase in Ca^2+^ influx through Ca_v_1.2 which in turns produces high levels of mitochondrial ROS. Thus, CaMKII was overactivated by a synergistic triggering by Ca^2+^ and ROS. The overactivation of CaMKII stimulated a remodeling of cardiac action potential able to induce arrhythmia [221]. CaMKII can be persistently activated through the oxidation of methionine 280/281 in its regulatory domain, independently of Ca^2+^ and calmodulin binding [222]. Interestingly, in mouse models of diabetes and dilated cardiomyopathy [223, 224], CaMKII was also a crucial mediator of ROS production, suggesting the possibility that CaMKII oxidation contributes to a ROS-induced ROS-positive feedback.

Another example of these cooperative effects mediated by ROS is represented by hydroxyeicosatetraenoic acid (20-HETE) which increases during ischemia-reperfusion. 20-HETE stimulates NOX-derived O_2_^•-^ production, which activates Ca_v_1.2 via a PKC-dependent mechanism in cardiomyocytes [225].

In cardiomyocytes derived by mdx mice, NOX2 induced ROS production which, in turn, increases CaMKII oxidation, thereby promoting aberrant SR Ca^2+^ release. Inhibition of CaMKII oxidation reduced intracellular Ca^2+^ levels preventing ventricular arrhythmia in the mouse model of Duchenne muscular dystrophy [226].

Also, Na^+^ overload can induce high mitochondrial ROS that trigger CaMKII oxidation, resulting in alterations of mitochondrial metabolism and Ca^2+^ handling by SR in rabbit cardiomyocytes [227]. Another regulator of this machine is angiotensin II that can activate both PKA and CaMKII via oxidation by NOX2, which results in disturbed Na^+^ and Ca^2+^ currents (via PKA) and enhanced diastolic SR Ca^2+^ leakage (via CaMKII) leading to the proarrhythmic phenotype. This was prevented by using inhibitors of PKA and CaMKII [228].

The release of Ca^2+^ from SR via RyR2 represents a crucial event to E-C coupling; thereby, RyR2 has multiple sites for regulating Ca^2+^ release by phosphorylation and/or interaction with Ca^2+^, Mg^2+^, ATP, CaM, and calstabin2. This regulation process of RyR2 can be also affected by the oxidation. Changes in the redox status of RyR2 affect thiol groups thus influencing channel functionality. Indeed, for its activation, RyR2 requires the oxidation of more than 7 thiols per subunit. In contrast, the oxidation of fewer than 5.5 thiols per subunit can occur readily without affecting RyR2 function [229]. Thus, ROS can increase the open probability of RyR2 which results in an increase in intracellular Ca^2+^ and, consequently, leads to a higher Ca^2+^ binding to myofilament TnC, improving the risk of arrhythmias [230].

Upon *β*-adrenergic stimulation, the increased RyR2 activity alters mitochondrial Ca^2+^ homeostasis and increases mitochondrial ROS production. This, in a vicious feedback cycle, exacerbates diastolic SR Ca^2+^ leak by increasing RyR2 oxidation, driving spontaneous Ca^2+^ release that is detrimental in cardiac disease [231].

During ischemia, there are important metabolic changes including an increase in the cytosolic NADH/NAD^+^ ratio which induces the production of O_2_^•-^ mediated by NADH oxidase. In this harmful process that alters Ca^2+^ content, the oxidation of RyR2 plays an important role. Indeed, an increase in the cytoplasmic NADH/NAD^+^ ratio depresses SR Ca^2+^ release in ventricular cardiomyocytes. This effect appears to be mediated by direct NADH inhibition of RyR2 channel activity and by indirect NADH inhibition (O_2_^•-^ mediated) [232].

In addition, ROS can induce an increase in RyR2 Ca^2+^ release also affecting triadin, calstabin2, and calsequestrin2, as well as indirectly both CaMKII and PKA [233–236].

During cardiac cycle after the systolic phase, there are four systems involved in the diastolic phase to remove Ca^2+^ from the cytosol: NCX, sarco-endoplasmic reticulum calcium ATPase (SERCA), Ca^2+^-uniport in the mitochondria, and sarcolemmal Ca^2+^-ATPase pumps. NCX and SERCA have a key role in the diastolic phase of the cardiac cycle, while the other systems have a minor role. All these systems interact to regulate the amount of Ca^2+^ within the cell at rest, most of which is stored within the SR [237].

NCX is a membrane transporter and NCX1 is the cardiac isoform. It contains ten transmembrane segments, and between segments 5 and 6, there is a large cytoplasmatic loop that plays a regulatory role [209, 238–240]. Ion transport is associated with two intramolecular regions called *α*1 and 2, located in transmembrane segments 2-3 and 7-8 [241]. NCX1 is placed widely on the sarcolemma but located preferentially in the T-tubules [242]. NCX1 is activated by Ca^2+^, while it is inhibited by Na^+^ [243, 244]. NCX1 uses an electrochemical gradient to release one Ca^2+^ ion and to get in the three cell Na^+^ ions. In detail, Na^+^ must be low in the cytosol for NCX1 activity. This is promoted by the Na^+^/K^+^ATP-dependent pump (Na^+^/K^+^ ATPase) that takes out Na^+^ entering the cell during action potential. If Na^+^/K^+^ ATPase is inhibited by digoxin or ouabain, accumulation of intracellular Na^+^ occurs and NCX1 reverses the flux of ions, increasing [Ca^2+^]_*i*_. This can lead to an enhanced cardiac contraction with the development of arrhythmia. So NCX1 has a critical role for the maintenance of ionic gradient between the extracellular space and cytosol; also, it is involved in action potential repolarization and duration [245]. In addition, NCX1 is implicated in the generation of the pacemaker potentials in the cells of the sino-atrial node. Thus, the spontaneous release of Ca^2+^ from SR activates NCX1 with a net inward ionic flow. This flux induces depolarization helping the pacemaker cells to reach the threshold to trigger the next action potential [246]. The reuptake of Ca^2+^ into the SR is mediated by the SERCA pump which uses the energy from ATP hydrolysis. There are three isoforms of this protein: SERCA1, SERCA2, and SERCA3, each isoform undergoes alternative splicing. Spliced isoforms of SERCA2 are SERCA2a and SERCA2b that differ in their affinity for Ca^2+^. SERCA2a is the isoform expressed in the cardiomyocytes while SERCA2b in all types of cells. SERCA2a is usually located in a region of SR separated from the junctional zone where there is RyR2 [247]. Two Ca^2+^ ions in the cytosol bind to SERCA2a determining its phosphorylation by ATP and, consequently, a conformational change triggering the uptake of Ca^2+^ into SR. When SERCA2a is dephosphorylated, it returns to the basal inactive state [248]. As SERCA2a activity promotes the removal of more than 70% of Ca^2+^ in the human heart, the function of SERCA2a is crucial in Ca^2+^ cycling [249]. Thus, it has a key impact for both the diastolic phase and the subsequent systolic force contraction induced by Ca^2+^ release from the SR. The main regulator of SERCA2a is phospholamban, a small, reversibly phosphorylated transmembrane protein, located in the cardiac SR. Dephosphorylated phospholamban binds to SERCA2a inducing an inhibitory effect. After the phosphorylation by protein-kinase A or CaMKII*δ*, phospholamban dissociates from SERCA2a allowing the pump activation [250]. This latter effect positively regulates the rate of cardiac relaxation, and on the subsequent beat, contractility is developed in relation to the entity of the SR-Ca^2+^ storing and SR-Ca^2+^ release. The dephosphorylation of phospholamban by protein phosphatase 1 allows its binding to the SERCA2a; thus, it is involved in the ending of the active phase of the pump [251].

Also, the alterations of proteins and channels acting during the diastolic phase play a critical role in heart diseases. The alterations of NCX1 expression are associated to higher [Ca^2+^]_*i*_ causing arrhythmia [252]. There are growing evidences that the drug inhibition of NCX1 may be an important therapeutic strategy, demonstrating the role of the exchanger in the genesis of arrhythmia [253]. In several studies of heart failure on animal models and humans, a decrease in phosphorylation of phospholamban and of SERCA2a activity has been observed. The reduction of phosphorylated phospholamban is one important factor determining the observed reduction in SR Ca^2+^ reuptake in the heart failure. These findings highlight the important role of SERCA2a and its regulatory proteins in the heart failure [254]. SERCA2a-KO mice exhibit inefficient Ca^2+^ handling, reduced contractile efficiency, and possible heart failure [255], while mutations in the phospholamban gene have been linked to hereditary cardiomyopathy and arrhythmias [256, 257]. On the other hand, in the ischemia/reperfusion injury model, SERCA overexpression is able to restore mitochondrial function by inhibiting Ca^2+^ overload, inactivating XO and thus reducing ROS.

It is not surprising that NCX1 can be oxidated by ROS that induce its activation [258]. In vitro, the treatment with H_2_O_2_ caused opposite effects on SERCA and NCX; the former is decreased while the latter is increased, leading to the redox-mediated SR Ca^2+^ depletion [259, 260]. This situation is common in heart failure, suggesting the huge impact of oxidative stress and its posttranslational mechanisms on health functioning of the heart. However, it remains controversial whether NCX itself is subjected to kinase regulation. In fact, while no functional change in NCX was observed on application of catalytic subunits of PKA and PKC [261], other authors showed that the intracellular loop of NCX can be phosphorylated by PKA or PKC, which may increase NCX activity contributing at list in part to the ROS-induced NCX activation [262].

SERCA2 contains 25 cysteine residues, but just a couple of them can be modulated by ROS inhibiting the activity of SERCA2 and reducing the Ca^2+^ uptake into the SR. Furthermore, ROS cause inhibition of SERCA uncoupling Ca^2+^ uptake activity from ATP hydrolysis [263, 264]. On other hand, ROS can act indirectly on SERCA modulation by activating protein kinases and partially counterbalancing impaired SR Ca^2+^ refilling by phospholamban phosphorylation by CaMKII at threonine 17 and PKA at serine 16. This results in phospholamban dissociation and activation of SERCA [265].

In summary, ROS increase the cytosolic Ca^2+^ and decrease its sequestration and extrusion. Ca^2+^ overloading leads to accelerating ROS production. In parallel, the rise of ROS drives to oxidize cysteine targets of various channels and pumps which regulate the contractile machinery. This triggers a positive feedback process which leads to the rise of intracellular Ca^2+^ and ROS. This crosstalk between Ca^2+^ and ROS is seen in physiological conditions to allow the proper cardiogenesis and E-C coupling, but when unbalanced, it has serious consequences leading to pathological conditions. Indeed, an increasing body of evidence suggests that high amount of ROS may promote directly or indirectly cardiovascular diseases, including cardiac hypertrophy, atherosclerosis, hypertension [266, 267], ischemia [268], diabetes [269], and heart failure [270].

## 5. Intracellular Zinc: Its Pivotal Role in Cardiomyocytes

### 5.1. An Overview of Intracellular Zinc Ion Dynamics

The human body contains about 2-3 g of zinc ion (Zn^2+^), and it is widely diffused in all tissues and organs even if it is in different concentrations. The human heart and blood plasma are known to contain 0.4% and 0.1% of total zinc, respectively [11]. Unlike other metals (such as iron Fe^3+^/Fe^2+^ and copper Cu^+^/Cu^2+^), Zn^2+^ is present as a stable divalent cation and does not directly undergo redox reactions, and for this reason, compounds containing Zn^2+^ are rare. On the other hand, Zn^2+^ is an efficient Lewis acid able to form coordination bonds often integrated with four bonds into a tetrahedral array with side chains of amino acids such as aspartic acid, glutamic acid, cysteine, and histidine [271, 272]. Intracellular Zn^2+^ exists in three forms: tightly bound to proteins, loosely associated with proteins or ligands, and free or labile Zn^2+^ [273]. The cellular content of Zn^2+^ is in the range of hundreds of micromolar, while the free Zn^2+^ ion concentration is in the picomolar range [274]. This difference depends mainly on the presence of intracellular specific Zn^2+^-binding proteins. Changes in [Zn^2+^]_*i*_ lead to activating cell signals. There are two forms of zinc signaling in the cells, “early” and “late” signaling. The early zinc signaling is independent of the gene transcription, and it is the result of rapid changes of [Zn^2+^]_*i*_ due to the Zn^2+^ release from cellular organelles into the cytosol. These changes take the name of “zinc waves,” and they occur in few minutes [275, 276]. The late zinc signaling is triggered by extracellular events that lead to gene transcription causing changes in proteins involved in zinc homeostasis [277]. It is necessary to highlight that the terms “early” and “late” do not refer to the Zn^2+^ kinetic, but they describe the downstream effects triggered by these two Zn^2+^ signaling patterns. Thus, the “Zn^2+^ waves” act as a second messenger, which is a fast intracellular signal, while the “late” Zn^2+^ is slower because it affects gene expression. In these two ways, intracellular Zn^2+^ is essential in cellular metabolism as a rapid signal as well as in differentiation and cell growth through a “late” signal.

In conclusion, Zn^2+^ not only is a structural component but also is involved in catalytic activation and intracellular signaling [273, 278]. Thus, Zn^2+^ is structurally part of approximately 3000 proteins, such as enzymes, transcriptional factors, and proteins involved in the DNA repair [279]. Moreover, Zn^2+^ facilitates catalytic modulation in several enzyme classes, such as oxidoreductases, hydrolases, and transferases. Finally, Zn^2+^ released in response to different stimuli acts as a signaling mediator and second messenger able to target several enzymes and proteins involved in cellular signaling, including channels, kinases, caspases, aconitase, and PKC [280].

**Figure 4:**
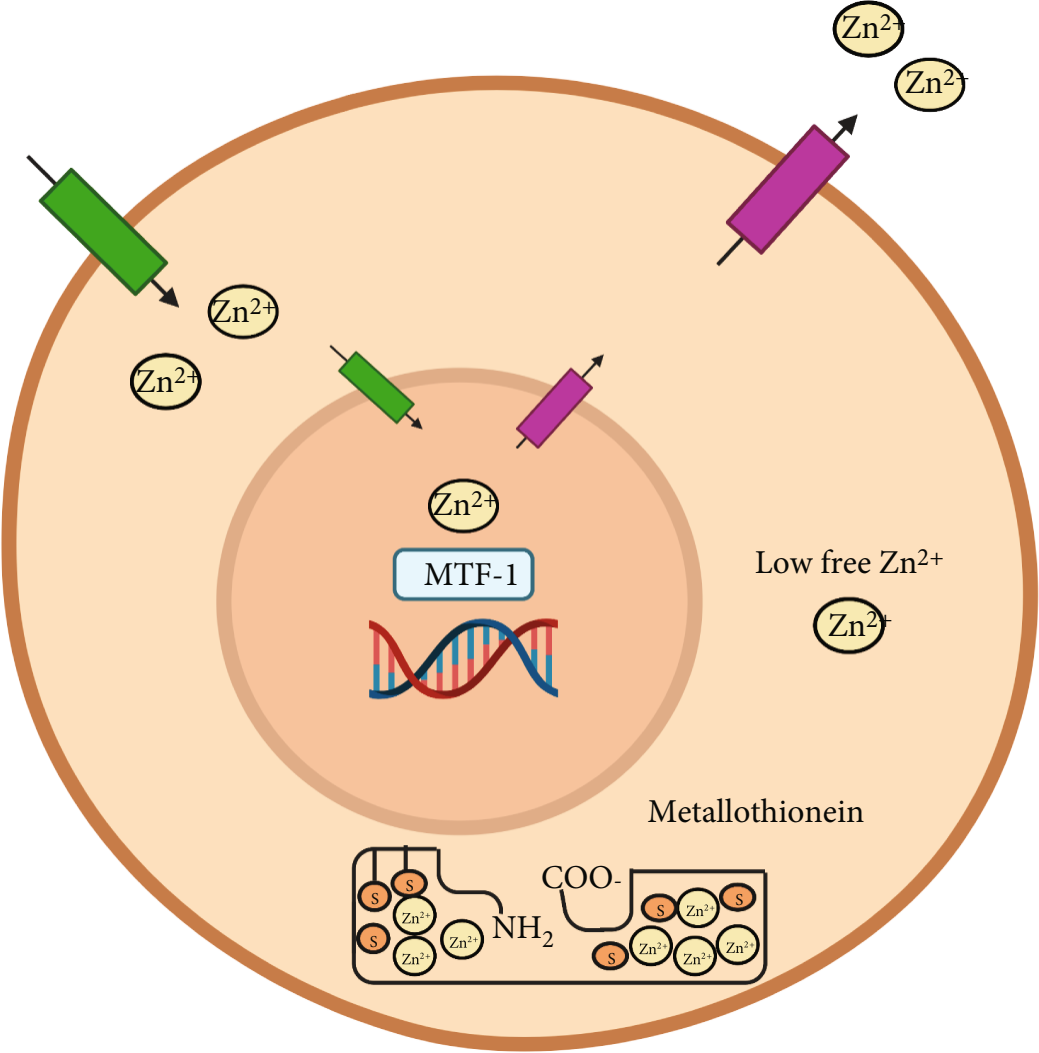
Actors controlling Zn^2+^ homeostasis.

To preserve a physiological balance of free [Zn^2+^]_*i*_, the Zn^2+^ homeostasis is tightly controlled at the whole body, tissue, cellular, and subcellular level by several proteins. In humans, the metal responsive transcription factor-1 (MTF-1), metallothioneins, and zinc transporters are involved in controlling cellular Zn^2+^ (Figure 4) [278]. In addition, there are other unconventional transporters involved in Zn^2+^ mobilization across the cellular membrane, including some type of voltage-gated Ca^2+^ channels and glutamate receptors [281].

MTF-1 is a zinc-dependent transcription factor which is involved in the regulation of intracellular signaling pathways. It is involved in the defense against oxidative stress, balance of trace elements (zinc, iron, and copper), inflammation, and proper fetal development. MTF-1 needs Zn^2+^ to translocate from the cytoplasm into the nucleus and to work as a transcriptional factor [282]. Activation of MTF-1 can take place directly by Zn^2+^ in the cytosol and indirectly by the release of zinc from metallothioneins or phosphorylation/dephosphorylation of zinc-binding proteins. Once MTF-1 translocates into the nucleus, it recognizes and binds to the specific region of the gene promoter, the metal-response element [283]. More of one thousand genes have the metal-response element, but at least forty-three genes have been identified as supposed targets of MTF-1. Among these targets, there are transcriptional factors involved in the proper development: glutamate-cysteine ligase, ZnT-1, and metallothioneins [284]. Oxidative stress induces the release of Zn^2+^ from metallothioneins and zinc-binding proteins, raising the [Zn^2+^]_*i*_, which in turn activates MTF-1. It induces the synthesis of metallothioneins and the expression of zinc transporters (such as ZnT-1) located in the cellular membrane. The metallothioneins bind to the excess of Zn^2+^, while ZnT-1 enhances the Zn^2+^ efflux. In this way, the cell reacts to high Zn^2+^ levels induced by ROS, by dampening [Zn^2+^]_*i*_ and restoring the physiological Zn^2+^ levels through the shuttling of the Zn^2+^ into subcellular stores (such as the endoplasmic reticulum, Golgi apparatus, lysosomes, and mitochondria) [142, 285–287] or by removing zinc ions' excess from the cell [288]. Moreover, Zn^2+^ can be accumulated also in intracellular vesicles for storage and/or release: these vesicles containing zinc are named zincosomes [289].

Metallothioneins are low molecular weight peptides rich in cysteine residues. Their expression is associated with toxicity caused by heavy metals and protection against DNA damage, oxidative stress, and apoptosis [290]. Metallothioneins are placed in various cellular compartments, such as the nucleus, cytosol, and cellular organelles. Human metallothioneins have a total of 11 functional isoforms that can be divided into four classes: designated metallothioneins 1, 2, 3, and 4. The most spread isoforms are metallothioneins 1 and 2 expressed in almost all tissues including the heart in which metallothionein 2A is the most abundant isoform [273]. The metallothioneins 3 and 4 are confined to the central nervous system and epithelium, respectively [291, 292]. Usually, the basal levels of metallothioneins 1 and 2 are very low, and their biosynthesis is inducible by several stress conditions and molecules, including glucocorticoids, cytokines, ROS, and metal ions (zinc and copper). Thus, the inducible expression of metallothioneins 1 and 2 is regulated by oxidative stress or metal-response elements which promote the nuclear translocation and activation of MTF-1. The metallothioneins 1 and 2 are involved in maintaining cell zinc homeostasis and buffering heavy metal-induced cytotoxicity by chelating these ions and lowering their intracellular free concentrations. Metallothioneins bind to Zn^2+^ more tightly than other zinc-binding proteins up to seven Zn^2+^ with different affinity [293]. This finding has significant implications for the function of metallothioneins because it demonstrates that metallothioneins are not a mere thermodynamic sink for Zn^2+^ but can participate actively in the process of cellular zinc redistribution. The unique coordination environment of metallothioneins allows redox mechanisms to control the availability of zinc. Indeed, cisteine sulfurs can undergo reversible redox reactions with concomitant release of Zn^2+^, thereby coupling redox reactions and zinc metabolism [294]. The release of Zn^2+^ from metallothioneins and other zinc-binding proteins mediated by ROS leads to the rise of [Zn^2+^]_*i*_: it represents a harmful intracellular signal. As mentioned above, the great amount of Zn^2+^ can bind and activate MTF-1 and, consequently, triggers the biosynthesis of inducible metallothioneins (metallothioneins 1 and 2) to scavenge ROS and decrease free Zn^2+^ in the cells [295–297]. Oxidative stress could activate MAPK subfamily members including p38, ERK, and JNK; whereas metallothioneins could exert inhibitory effects of MAPKs regulating the pathogenesis of diseases based on oxidative unbalance [298]. In addition, after treatment with cadmium, that induce apoptosis, metallothionein^−/−^ cells showed significantly higher increases in phosphorylated JNK than metallothionein^+/+^ cells, this is consistent with “in vivo” data revealing a higher increase in phosphorylated JNK1/2 levels in cadmium-treated metallothionein-null mice compared to wild-type mice [299]. However, the detailed molecular mechanisms that link zinc and MAPKs have not been defined. Thus, the overexpression of metallothioneins represents an effective defense strategy of cells to reduce ROS and DNA damage, as shown in *β* cells of streptozotocin-induced diabetic mice preventing diabetic symptoms, in cardiomyocytes preventing diabetic cardiomyopathy [300, 301] and in ischemia-reperfusion conditions [302]. Indeed, polymorphisms of metallothionein 2A gene are associated with ischemic cardiomyopathy in diabetic patients and susceptibility of atherosclerosis [303, 304]. Overall, metallothioneins are highly dynamic proteins and they draw a strong link between zinc homeostasis and redox status and, for this, play a crucial role to balance intracellular Zn^2+^ in physiological and stress conditions.

Zinc transporters are divided in two classes: 10 zinc transporter proteins (ZnT), which are mostly located on intracellular membranes and export Zn^2+^ from the cytosol, and 14 Zrt- and Irt-like proteins (ZIP) which are mostly located on the plasma membrane and import Zn^2+^ into the cytosol. The activation of ZnT and ZIP transporters is mediated by zinc coordination at the enzyme active site and some transporters mobilize not only zinc, but also iron, manganese and cadmium [295]. The expression of ZnT and ZIP transporters is sophisticatedly coordinated by transcriptional and posttranscriptional regulations, including transcriptional activation, mRNA stabilization, protein modifications, trafficking to target organelles, and degradation, in response to various stimuli, including hormones, cytokines, endoplasmic reticulum stress, oxidative stress, and hypoxia. All these mechanisms are handled in a cell- and tissue-specific or differentiation- and developmentally regulated manners. The rapid Zn^2+^-responsive transcriptional control of some ZnT transporters, as ZnT-1 and ZnT-2, requires MTF-1 [305]. Commonly, ZIP transporters constitute homodimers or heterodimers with eight transmembrane domains [190, 306, 307], while ZnT transporters, except for the heterodimer ZnT5 and ZnT6 [308], form homodimers with six transmembrane domains [309]. ATP hydrolysis is not required for Zn^2+^ mobilization across the biological membrane. ZIP transporters may function as selective electro-chemical diffusion channels [310], or as zinc/bicarbonate symport transporters [311, 312]. On the other hand, Zn^2+^ mobilization by ZnT proteins is thought to be dependent on the proton electrochemical gradient, and the zinc-binding site of ZnT proteins is essential for Zn^2+^ transport [313–315].

Like metallothioneins, also zinc transporters are very redox-sensitive targets. Indeed, the protein levels of ZIP5, ZIP7, ZIP14, and ZnT7 were affected by H_2_O_2_ and 4-HNE (one metabolite of lipid peroxidation). 4-HNE significantly decreased ZIP5 levels and increased the ZIP7 levels; whereas, H_2_O_2_ remarkably reduced ZIP14 protein expression and elevated the ZIP7 and ZnT7 protein expressions [316]. In streptozotocin-induced diabetic rats, zinc dyshomeostasis was observed according with perturbations of zinc transporters levels in body tissue, including increased ZnT5 expression in the heart. Dietary zinc supplementation leads to beneficial effect in the control of diabetes-induced zinc dyshomeostasis. This is achieved through regulation of the tissue specific zinc transporters along with stimulation of metallothionein synthesis that scavenges oxidative stress induced by diabetes itself [317].

An increasing body of findings demonstrated that the main isoforms of ZIP and ZnT transporters expressed in the human heart are ZIP 1, ZIP 6, ZIP 7, ZIP 9, ZIP 13, and ZIP 14 and ZnT 1, ZnT 5, ZnT 7, and ZnT 9, respectively [273, 318, 319]. The expression levels of these isoforms depend on physiological or pathological conditions. In a recent study, these and other isoforms of ZIP and ZnT transporters have been identified in adult rat ventricular cardiomyocytes and their expression changes linked to extracellular Zn^2+^ or to the presence of TPEN, a strong heavy metal chelator [320].

Table 1 summarizes the consolidated evidences from different studies demonstrating the alterations of zinc transporters that lead to unbalanced zinc homeostasis which, in turn, promotes the onset of several cardiac diseases and other pathological conditions.

Hence, well-controlled [Zn^2+^]_*i*_ homeostasis occurs through a fine coordination among metallothioneins and ZIP and ZnT transporters. Any perturbation of these control systems induces the impairment of zinc homeostasis [321].

Mild zinc deficiency may lead to the dysfunctions of the immune response, brain, sensory organs, reproductive system, and cardiovascular activity. Low level of zinc in the body, and/or individual tissues, can raise cardiovascular risk [322, 323]. It is necessary that the systems controlling zinc homeostasis work together to ensure physiological levels of intracellular Zn^2+^ which is essential in human health, especially in terms of antioxidant and anti-inflammation responses [324].

As an antioxidant, Zn^2+^ is a redox-inactive ion, but, in contrast to magnesium and calcium, Zn^2+^ can interact with the sulfur (thiolate) donor ligand of cysteine, forming Zn^2+^-thiolate which can be redox-active. This peculiar Zn^2+^ feature links Zn^2+^ to the cell oxidative status. ROS and oxidants can interact with thiolate and induce the release of Zn^2+^ in a free state. Free Zn^2+^ acts as a signaling element that triggers indirectly an antioxidant response through different pathways. These indirect effects occur only at certain concentrations because the zinc deficiency or overload produces a prooxidant effect [325].

Most of Zn^2+^-related effects are also due to extracellular Zn^2+^ that acts through a G-protein coupled receptor (GRP39). The binding of Zn^2+^ to GPR39 was seen in different cell phenotypes and triggers G-protein-dependent intracellular signaling related to ion transport mechanisms, cell growth and survival [326].

The main zinc-mediated antioxidant and anti-inflammation pathways are summarized in Tables 2 and 3, respectively [327].

Deficient or excessive Zn^2+^ concentrations can result as prooxidant and proinflammatory factors. The binding between Zn^2+^ and metallothioneins is more sensible to oxidative stress that causes the release of Zn^2+^. This leads to an increase in free Zn^2+^ that enhances oxidative stress and promotes cell death. These conditions are associated with several cardiovascular diseases, including diabetes, atherosclerosis, heart failure, and hypertension [12, 273].

### 5.2. Focusing on the Role of Zinc Ion in Cardiomyocytes

To date, regarding the role of Zn^2+^ in the cardiovascular physiology, little is known about the molecular mechanisms of Zn^2+^ signaling. In the heart, the zinc homeostasis allows the proper balance of ROS and cardiac function. Zinc deficiency leads to the impaired E-C coupling, an increase in ROS production and damage (as the enhanced lipid peroxidation), associated with a decrease in antioxidant defense of cardiomyocytes. On the contrary, the excess in Zn^2+^ promotes the increase in inflammatory pathways and phosphorylation of CaMKII, PKA and RyR2. In addition, the overload of Zn^2+^ induces a Ca^2+^ dyshomeostasis, which, in turn, leads to impaired E-C coupling and mitochondrial dysfunction with the rise of ROS levels, apoptosis, and cardiomyocytes' death [328].

Cardiomyocytes have a little but detectable free Zn^2+^ pool of about 100 pM [329], and small changes of [Zn^2+^]_*i*_ can lead to a marked effect on cardiac function [330].

When exposed to extracellular zinc, isolated rat and mouse cardiomyocytes showed a significant elongation of relaxed sarcomere length, reductions in intracellular Ca^2+^ peaks, SR Ca^2+^ loading, and Ca_v_1.2 channel inward currents. In addition, after perfusing the heart with 50 *μ*M zinc, a marked dephosphorylation of serine 2808 in RyR2 and of serine 16 and threonine 17 in phospholamban was found. These data collectively suggest that the exposure to zinc resulted in inhibiting Ca^2+^ rise thus reducing cardiac contractility and enhancing relaxation [331]. Indeed, the phosphorylation of serine 2808 in RyR2 is associated with an increased channel open probability and SR Ca^2+^ leak due to the dissociation of calstabin2 [332]. The Zn^2+^-mediated reduction of phosphorylated RyR2 and consequent cardiomyocyte relaxation were more evident in rat cardiomyocytes upon hyperglycemia condition [331, 333]. This could be explained considering that RyR2 phosphorylation was found enhanced in pathological conditions like hyperglycemia [333] and diabetes [334]. Under pathological stimulus, such as hyperglycemia, an increased production of RNS besides ROS was observed, which in turn induced marked increases in [Zn^2+^]_*i*_ level via either mobilization of Zn^2+^ from metalloproteins or release of Zn^2+^ from SR or both ways. Consequently, this increased [Zn^2+^]_*i*_ can induce phosphorylation/oxidation of RyR2 due to a higher level of phosphorylated protein phosphatases, which induce also further increases in [Zn^2+^]_*i*_ [335]. Other findings showed that the exposure to extracellular zinc reduced Ca^2+^ influx through Ca_v_1.2 channels and this effect was reverted by zinc washout [336]. Indeed, these studies confirmed the role of Zn^2+^ in modulating current passage through many ion channels. Also, Ca_v_1.2 channels are modulated by extracellular or intracellular Zn^2+^ depending on Zn^2+^ itself and Ca^2+^ concentrations. In addition, intracellular Zn^2+^ (at about 1 nM) inhibits also *β*-adrenergic stimulation, probably altering adenylyl cyclase activity. This effect may account, at least in part, the reduced *β*-adrenergic stimulation generally reported in the cardiopathies that show an increased basal Zn^2+^ concentration [337].

The adrenergic system is very important in the heart and can modulate both Ca^2+^ and Zn^2+^ levels. Indeed, a single infusion of isoproterenol, a synthetic catecholamine, induces alterations of extra- and intracellular Ca^2+^ and Zn^2+^ levels in different tissues, including the myocardium. The increase in intracellular Ca^2+^ leads to a high ROS production and opening of mitochondrial permeability transition pore. Simultaneously, it was observed a decrease in intracellular Zn^2+^ that contributes to exacerbate this oxidative stress. All together lead to cardiomyocytes' necrosis and fibrosis [338]. These findings were supported using *β*-adrenergic receptor antagonists, such as carvedilol and timolol or quercetin, a flavonoid with antioxidant properties. These molecules normalize Ca^2+^ and Zn^2+^ levels and avoid the damage of cardiomyocytes [338, 339].

Considering all these evidences, under several pathological conditions, increased [Zn^2+^]_*i*_ in cardiomyocytes is closely responsible for alterations in proteins (RyR2 and Ca_v_1.2 channels) and for production of intracellular ROS and RNS, which in turn induce higher levels of phosphorylation and oxidation in the contractile machinery.

For the first time, Woodier and colleagues demonstrated that Zn^2+^ can modulate RyR2 also in a Ca^2+^-independent manner. Testing different Zn^2+^ concentrations on the cytosolic face of RyR2 channels incorporated in liposomes, Zn^2+^ raised the opening probability of RyR2 in a dose-dependent response from 100 pM up to 1 nM. Zinc concentrations in the range of 1-10 nM did not cause any significant effect on the RyR2 gating. The addition of 1 mM zinc completely inhibited the channel opening. These suggest that there are low and high affinity specific sites on RyR2 that binds zinc. To test the impact of Zn^2+^ on Ca^2+^ spikes, isolated cardiomyocytes were perfused with different Zn^2+^ concentrations (100 pM - 10 nM). This experimental condition caused an increase in both frequency and amplitude of Ca^2+^ spikes in a zinc concentration-dependent manner. On the other hand, the presence of extracellular zinc concentrations greater than 100 nM induced a decrease in the amplitude and frequency of Ca^2+^ spikes [340].

The growing interest in Zn^2+^ signals is an incentive to acquire a better understanding of the molecular mechanisms of the Zn^2+^ signals. Therefore, the discovery that Zn^2+^ can modulate RyR2 in a Ca^2+^-independent manner opens the way to the study of different pathophysiological consequences in the cardiovascular system. In fact, small variations of free intracellular Zn^2+^ can modify the opening of RyR2 with deleterious effects. In pathological conditions high [Zn^2+^]_*i*_ is observed (may reach up to 1 nM), and Woodier and colleagues proposed that zinc is the main activating ligand of RyR2 with a higher affinity than calcium. This leads to an increase in SR Ca^2+^ leak and of [Ca^2+^]_*i*_ due to long-lasting open states of RyR2 that cause arrhythmia and dysfunction in heart failure [340]. As cited above, the main factor involved in the rise of intracellular free Zn^2+^ is the oxidative stress through the oxidation of the cysteine groups of metallothioneins and zinc-binding proteins. Indeed, it is not a coincidence that high ROS levels in cardiomyocytes are associated with several cardiovascular diseases.

Altered intracellular and extracellular Zn^2+^ levels also affected cell membrane turnover and mitochondria that show different morphological and functional alterations. In rat cardiomyocytes, a high intracellular Zn^2+^ increased cell surface areas, mimicking a cardiac hypertrophy-like process. On the other hand, the increased extracellular Zn^2+^ caused a decrease in cardiomyocytes surface area. The rise of [Zn^2+^]_*i*_ has resulted in degradation of myofibrils and in mitochondrial damage through defects in fission-fusion dynamics. Thus, high [Zn^2+^]_*i*_ leads to increase different markers of SR stress, like calregulin and glucose-regulated protein 78, and NF-*κ*B phosphorylation, which in turn induces ROS production and apoptosis [341]. In other conditions, Zn^2+^ was necessary to support the cardioprotective effects induced by inhibitors of SR stress or antioxidant molecules [342, 343]. Zinc administration inhibits the opening of mitochondrial permeability transition pore through the injury salvage kinase (RISK) pathway activation [344]. Zinc allows the phosphorylation of the components of RISK pathway: PI3K/Akt [345], ERK1/2 [346], and downstream target glycogen synthase kinase-3 beta (GSK-3*β*) [347], thus contributing to RISK activation. Also, in H9C2 cells, exogenous Zn^2+^ inhibited protein phosphatase 2A promoting RISK pathway activation and the inhibition of mitochondrial permeability transition pore. In addition to what mentioned above, zinc deficiency in rats enhances myocardial injury through depletion of GSH, associated with oxidative-induced damage in ischemia [348]. The pre-treatment of rats with zinc and acetylsalicylic acid complex is more efficacious than acetylsalicylic acid alone in protecting the heart from acute myocardial ischemia. This effect is mediated by Zn^2+^-improved activity of antioxidant enzymes, as SOD1 and glutathione peroxidase, reducing proinflammatory prostanoids involved in the development of ischemia [349].

Furthermore, cardiac mitochondria produce nitric oxide that mobilizes both intracellular Zn^2+^ via cGMP-protein kinase G signaling and intracellular Ca^2+^ that may result from Ca^2+^ uptake by the Ca^2+^ uniporter [346, 350].

In addition, also ROS promote nitric oxide generation from inducible nitric oxide synthase which in turn promotes the release of Zn^2+^ from metallothioneins [351].

Cardiometabolic diseases, like diabetes and atherosclerosis, are always accompanied by altered zinc homeostasis and systematic inflammation. Low Zn^2+^ levels facilitate ROS production and an increase in NF-*κ*B and release of inflammatory cytokines. On the other hand, these cytokines generally induce a downregulation of zinc transporters, suggesting that chronic inflammation can perturb zinc homeostasis [352]. In these pathological conditions, intracellular levels of GSH and metallothioneins are very low and the zinc supplementation in vitro and in vivo prevents the development of cardiomyopathy through the synthesis of metallothioneins which exert their antioxidant function [353].

Aldosteronism is associated with dyshomeostasis of Zn^2+^ and Ca^2+^ that contributes to heart failure, a chronic progressive condition that affects the pumping power of heart muscle. Zinc depletion in plasma and intracellular storages accompanied by reduction of metallothioneins and SOD synthesis were observed in patients with heart failure. All of these, in turn, promote oxidative stress in heart failure. In addition to Ca^2+^ and Mg^2+^ leaks, the principal underlying pathophysiologic mechanism for hypozincemia is the renin-angiotensin-aldosterone system (RAAS). The activation of RAAS induces an increase in fecal and urinary zinc excretion that is blocked by antagonists on aldosterone receptor. Other factors involved in hypozincemia in heart failure include urinary zinc excretion associated with angiotensin-converting enzyme inhibition treatment, reduced dietary zinc intake and/or impaired small intestinal absorption of zinc or its increased uptake in stressed tissue [354]. Aldosterone/salt treatment and parathyroid hormone lead to an increase in intracellular and mitochondrial Ca^2+^ which ensures ROS. Together with membrane potential lost and ATP synthesis depletion, the pathological opening of mitochondrial permeability transition pore follows to cardiomyocytes necrosis with leakage of troponins. To reverse this prooxidant phenotype observed in heart failure, it was necessary the administration of zinc, calcium and magnesium, which reduced ROS production and preserved mitochondria [322].

Both zinc deficiency and excess are detrimental to cells, causing impaired E-C coupling in cardiomyocytes [355] and apoptosis with the dissipation of mitochondrial membrane potential that leads to the release of cytochrome c [356]. Zinc dyshomeostasis occurs spontaneously to regulate oxidative stress in cardiomyocytes. This is always coupled to an increase in antioxidant defenses to fight prooxidant effects. However, beyond a certain threshold, the balance shifts to the prooxidant phenotype which underlies on cardiovascular diseases. To date, the impact of zinc alterations (both deficiency and excess) on the development and progression of cardiovascular diseases is well accepted. Zinc alterations increase systemic inflammation, ROS levels with remarkable degeneration of cardiomyocytes, extensive areas of fibrosis and degradation of the collagenous scaffolding that normally provides support and maintains myocardial geometry during the cardiac cycle. To counteract these deleterious effects mediated by zinc alterations, a zinc supplementation in the diet was proposed in patients affected by cardiovascular diseases. Kamalov and colleagues suggested that an optimal intracellular Zn^2+^/Ca^2+^ ratio in cardiomyocytes and mitochondria may be relevant to scavenge oxidative stress. They reported that increased [Ca^2+^]_*i*_ and mitochondrial [Ca^2+^] were linked to the induction of oxidative stress, while antioxidant effects resulted from the rise in [Zn^2+^]_*i*_ and mitochondrial [Zn^2+^] accompanied by a simultaneous activation of MTF-1 and induction of metallothionein-1 and glutathione peroxidase [357]. This is an important issue for future research to evaluate the hypothesis of cardioprotective effects of zinc supplementation, nevertheless randomized trials are needed.

## 6. Conclusions

The heart is one of the highest energy-demanding organs with a high number of mitochondria essential to produce ATP and ensure cardiac contraction. Nevertheless, these important organelles are also the main sources of ROS, whose altered handling can cause their accumulation and therefore triggers detrimental effects on mitochondria themselves and other cell components thus leading to apoptosis and cardiac diseases [358, 359].

Here, the signaling of ROS can shift rapidly between physiological and pathological status depending on the type of ROS, their concentration, and production sites. It is also interesting to evaluate the clinical significance that can be derived from these studies. In fact, understanding the molecular and physiological aspects of Zn^2+^ homeostasis and its relationship with intracellular Ca^2+^ can be of great help in understanding cardiac pathophysiology.

Although each system seems to be independent and finely controlled, the contractile proteins, intracellular Ca^2+^ homeostasis, and intracellular Zn^2+^ signals are strongly linked to each other by the intracellular ROS management in a fascinating way to form a “tetrad” which ensures the proper functioning of the myocardium. Nevertheless, if ROS balance is not properly handled, this can affect one or more of these essential components altering their function; this results in deleterious chain reactions which lead to an unbalance of this “tetrad” and promoting cardiovascular diseases.

In conclusion, this “tetrad” is proposed as a complex network that communicates continuously in the heart and can drive the switch from physiological to pathological conditions.

## Figures and Tables

**Table 1:** Zinc transporters and their functions in cardiomyocytes.

Zinc transporters	Proposed pathways	Effects	References
ZnT-1	Overexpression of ZnT-1 reduces caspase activation	Cardioprotective effect in HL-1 cells	[360]
	During hypoxia, ZnT-1 expression increases and intracellular Zn^2+^ rises	Cardioprotective effect in myocardial ischemia-reperfusion model	[361, 362]

ZnT-5	Some stress proteins (c-fos and Cry61) are downregulated in hearts of Znt-5-mutant mice	Maturation of osteoblasts and maintenance of the cells involved in the cardiac conduction system	[363]

ZnT-7/ZIP7	In hyperglycemia condition in vitro, Zn^2+^ increases due to changes in the expression levels of transporters (ZIP7 and ZnT7) and metallothioneins	Dyshomeostasis of Zn^2+^ can affect hyperglycemia/diabetes-associated cardiac dysfunction	[14, 364, 365]

ZnT-8	In pancreatic *β*-cells of mouse, both deletion and overexpression of ZnT-8 induce alteration of Zn^2+^ and insulin secretion	Controlling Zn^2+^ and insulin release	[366–372]

ZIP2	In the presence of Zn^2+^ leak, the signal transducer and activator of transcription 3 (STAT3) induces the overexpression of ZIP2 which, in turn, increases cellular Zn^2+^ uptake	Cardioprotective mechanism in response to ischemia/reperfusion injury	[373]

ZIP8	In ZIP8-knockout mice, impairment of heart formation is proven	Endomyocardial trabeculation remodeling	[374]

ZIP14	ZIP14-null mice show low cytosolic Zn^2+^ levels, hyperinsulinemia, increased body fat, and increased proinflammatory pathways	Glucose and insulin level control. Alterations of ZIP14 can lead to diabetes	[375–378]

Zinc transporters in rat hypertrophic hearts	Alterations of zinc transporters lead to [Zn^2+^]_*i*_ increase, SR-Zn^2+^ leak, mitochondrial ROS production, and apoptosis	Differential changes in the expression levels of zinc transporters can promote hypertrophic condition of the heart via increased [Zn^2+^]_*i*_	[379]

Zinc transporters in heart failure	Alterations of zinc transporters' expression induce phosphorylation/activation of PKC, increase in [Zn^2+^]_*i*_ and ROS production, and apoptosis in cardiomyocytes	The alteration of zinc homeostasis can promote heart failure	[380]

**Table 2:** Main zinc-mediated antioxidant pathways.

Antioxidant pathways	References
Binding to cysteines, Zn^2+^ protects proteins by oxidation	[381]
Zn^2+^ binds to and activates MTF-1, promoting the expression of metallothioneins, ZnT-1, and other target genes (like selenoprotein-1, which encodes an antioxidant glutathione-binding protein)	[284]
Zn^2+^ binds to Keap1 inducing the release and nuclear translocation of Nrf2 to activate the metal-response element	[381]
Zn^2+^ is a structural component of antioxidant enzymes (like SOD) and affects glutamate-cysteine ligase expression thus increasing GSH production	[382]
Zn^2+^ competing with other metals, such as iron and copper, at the binding sites modulates NOX activity	[383]
Zn^2+^ enhances the glycemic control and insulin sensibility contributing to the decrease in ROS production under hyperglycemic conditions	[384]

**Table 3:** Main anti-inflammation pathways.

Anti-inflammation pathways	References
Zn^2+^ modulates NF-*κ*B activation regulating the release of cytokines and inflammatory processes	[273, 385–388]
Zn^2+^ regulating the translocation and activity of hypoxic-inducible factor-1 *α* (HIF-*α*) influences inflammatory cytokines' release and oxidative stress	[273, 352]
Zn^2+^ is involved in modulating the activity of PPAR agonists and the anti-inflammatory markers	[389, 390]

## Data Availability

The literature used to support the findings of this review is listed within the article (bibliography).
